# Spatial patterns of historical crop yields reveal soil health attributes in US Midwest fields

**DOI:** 10.1038/s41598-024-51155-y

**Published:** 2024-01-03

**Authors:** Ames Fowler, Bruno Basso, Fidel Maureira, Neville Millar, Ruben Ulbrich, William F. Brinton

**Affiliations:** 1 Department of Earth and Environmental Sciences, Michigan State University, 288 Farm Lane, East Lansing, MI 48823 USA; 2 W.K. Kellogg Biological Station, 3700 E. Gull Lake Dr. Hickory Corners, Michigan, MI 49060 USA; 3 Woods End Laboratories, 290 Belgrade Rd, Mt Vernon, Augusta, ME 04352 USA

**Keywords:** Agroecology, Carbon cycle

## Abstract

Attaining high crop yields and increasing carbon storage in agricultural soils, while avoiding negative environmental impacts on water quality, soil erosion, and biodiversity, requires accurate and precise management of crop inputs and management practices. The long-term analysis of spatial and temporal patterns of crop yields provides insights on how yields vary in a field, with parts of field constantly producing either high yields or low yields and other parts that fluctuate from one year to the next. The concept of yield stability has shown to be informative on how plants translate the effects of environmental conditions (e.g., soil, climate, topography) across the field and over the years in the final yield, and as a valuable layer in developing prescription maps of variable fertilizer rate inputs. Using known relationships between soil health and crop yields, we hypothesize that areas with measured constantly low yield will return low carbon to the soil affecting its heath. On this premises, yield stability zones (YSZ) provide an effective and practical integrative measure of the small-scale variability of soil health on a field relative basis. We tested this hypothesis by measuring various metrics of soil health from commercial farmers’ fields in the north central Midwest of the USA in samples replicated across YSZ, using a soil test suite commonly used by producers and stakeholders active in agricultural carbon credits markets. We found that the use of YSZ allowed us to successfully partition field-relative soil organic carbon (SOC) and soil health metrics into statistically distinct regions. Low and stable (LS) yield zones were statistically lower in normalized SOC when compared to high and stable (HS) and unstable (US) yield zones. The drivers of the yield differences within a field are a series of factors ranging from climate, topography and soil. LS zones occur in areas of compacted soil layers or shallow soils (edge of the field) on steeper slopes. The US zones occurring with high water flow accumulation, were more dependent on topography and rainfall. The differences in the components of the overall soil health score (SHS) between these YSZ increased with sample depth suggesting a deeper topsoil in the US and HS zones, driven by the accumulation of water, nutrients, and carbon downslope. Comparison of the field management provided initial evidence that zero tillage reduces the magnitude of the variance in SOC and soil health metrics between the YSZ.

## Introduction

Agriculture faces three major challenges: feeding an increasing human population, helping mitigate climate change, and reducing environmental damage due to water pollution, soil erosion, and biodiversity losses^1,2^. Agricultural soils play a vital role in providing ecosystem services by storing and transforming soil organic carbon (SOC), nutrients, and water while provisioning food, fuel, and fiber^3^. The ability of soils to efficiently convert irregular inputs of water and nutrients into stable, plant-available resources determines a soil’s productivity potential^4^ and is closely tied to a common definition of soil health: “the continued capacity of soil to function as a vital living ecosystem that sustains plants, animals, and humans”^5^. Measuring soil health has been grounds for a wide debate^6^.

While there is no universal measure of soil health, the literature broadly agrees that it encompasses chemical, physical, and biological attributes^7,8^, and that SOC is considered a key variable strongly and positively correlated with various soil health indicators^7–11^. Soil health testing grew out of efforts to evaluate the impacts of intensive farming on soil properties not routinely tested^12–15^. Parameters that characterized the biological impacts of farming included carbon respiration, organic-N mineralization, and the ratio of mineralized-N to organic-C^16,17^. Soil quality indexes are formed from the combinations of biological and chemical metrics, such as pH, organic matter, microbial biomass C, and respiration or enzyme activities^18–22^. The USDA has codified selected methods as recommended practice for laboratories^23^, and public and private soil testing organizations have selected variations of these indicators, and scores calculated from them, to complement soil chemical nutrient analysis. These include measures of aggregation stability, soil compaction and bulk density, SOC and it’s pool fractions, nutrient availability, and microbial activity^8,12,24–26^.

Several soil health indicator tests have been developed, including Alabama, Cornell, Haney, and Solvita. These tests, popular with producers, focus primarily on providing recommendations for within-year nutrient applications and generate a scaled, numerical value of soil health^27^. Increasingly used, these tests face debate over their consistency and repeatability^28,29^. While soil health literature focuses on the dynamics of SOC pools and the characteristics of soil food webs^7^, the variability in soils’ provisioning is also strongly controlled by the factors of soil formation; climate, parent material, topography, time, and organisms, including humans^30,31^.

Soil formation factors, and their relationship with soil health and SOC, control soil provisioning at different spatial and temporal scales. Adhikari et al., (2020) evaluated the relationships between environmental variables and SOC at local (100m) to regional (50km) scales^32^. They found that topography controls SOC heterogeneity locally while climate variables are more dominant regionally. In our study, we refer to these scales to help differentiate between regional factors that are effectively constant across a field, and local factors that vary substantially within a field. Climate, parent material, and time controls the weathering of rock to soil, and the associated accumulation of SOC at large spatial and temporal scales^9,33^. Enhanced rock weathering aside^34^, agronomic management has limited potential to quickly impact soil texture, minerology, or climate driven, carbon assimilation-decomposition rates which correlate with SOC at a regional scale^32,35,36^. At the local scale, soil and SOC particles flow down slope, leading to deeper and more SOC rich soils along a hillslope profile^37^. While agricultural management limits plant-soil interactions that are observed in natural environments, greater crop growth in down slope positions, as a result of water and nutrient accumulation, returns more biomass to the soil producing a secondary, reinforcing plant-soil feedback^10,38,39^. It is challenging to separate complex, subtle plant-soil interactions from the effects of topography and management history that drive soil formation^40^. However, when changes in soil health and SOC due to human management are evaluated over time^6^, the impact of formation factors on soil heterogeneity must be considered to help constrain the spatial variability.

Improving soil health with the accumulation of SOC increase the nutrient and water holding capacity of the soil through changes in structure and chemistry, which in turn increase the yield potential of the soil^41,42^. Precision agriculture increases efficiency and decreases pollution by quantifying and partitioning the in-field heterogeneity of crop yield potential into site specific management zones. Ignoring this heterogeneity leads to non-point source pollution and the expansion of agriculture into marginal and ecologically sensitive lands^43^. For example, in the USA Midwest, areas of low and stable (LS) crop yield leach 86% more nitrogen (N) when compared to areas of high and stable (HS) crop yield^44^. More precise N fertilizer management within a field using techniques such as variable rate nitrogen (N) applications, can increase fertilizer use efficiency, thereby reducing nutrient pollution, greenhouse gas emissions, and farming costs^45,46^.

Agricultural soils carry an estimated carbon debt of 133 Pg C^47^. Increases in SOC can deliver societal value through carbon re-sequestration in the soil and as a mid-term buffer as economies de-carbonize to address climate change. Implementing restorative practices such as land reversion to perennial cover, incorporation of high carbon concentration amendments, and adoption of no-till management and cover crops could accumulate SOC at rates of 0.6 to 1.2 Pg C yr^-1^ over a 50-year window^48,49^. As governments and private companies commit to net-zero carbon goals through routes that include SOC sequestration, carbon markets have emerged to help facilitate those investments. These markets need accurate measurement, reporting, and verification (MRV) of the changes in agricultural SOC stocks brought about by management to ensure integrity and increase the future value of the carbon credits traded^50,51^.

To help maximize SOC sequestration potential and incentivize the use of precision agricultural technologies, within field management zones can be used to account for soil heterogeneity and better enable efficient SOC sample stratification^52–54^. These zones are typically defined through an intensive and extensive soil sampling campaign and then extrapolated to larger spatial scales using models. This initial, comprehensive sampling is often prohibitively expensive for frequent and larger scale use, whereas less intense, limited sampling campaigns risk insufficient statistical power to detect management differences^51,55^. Constraining the spatial heterogeneity of the initial conditions of SOC is therefore needed for a cost-effective, and targeted assessment of future SOC changes^56^.

A reliable representation of soil heterogeneity can be gained from long-term crop performance as captured by spatial patterns of yield stability. Yield stability can be determined from data obtained directly from in-field crop yield monitors or indirectly via remote sensing technologies^33,44,57,58^. These data enable the calculation of field relative yield stability zones (YSZ) and the relation of yield data to remotely sensed vegetative indices^59^. By accumulating spatial data over multiple years and analyzing each year’s data relative to the field mean value, YSZ offer a valuable quantitative metric for integrating the long-term response of the plant to the underlying soil resource and its local variability^33^.

There are two key measures of plant growth that define YSZ: yield level and yield stability. The yield level represents the long-term, average, gridded yield relative to the field average, and can be categorized as high, medium, or low^60,61^. Yield stability is determined by the standard deviation of each grid cell's yield relative to the field average, and indicates whether the cell is stable or unstable. Previous works, analyzing 5520 crop years of data across 768 fields across the USA Midwest found that yield distribution is asymmetric, and that low yielding areas are lower in frequency but cover a larger range of low values^33,44,57,61^. Stable yield zones are those parts of the field that show consistent yield over time with the low variation attributable to year-to-year weather fluctuation, whereas unstable (US) zones show variable yield with year-to-year variations in weather conditions. The US zones tend to occur in areas of the field that receive excessive or insufficient water, such as the shoulder and back slope of elevated areas, or in depressions^62^.

Because YSZ represent a spatially continuous, high resolution (2 m from yield monitors^57^—30 m from Landsat remote sensing^44^) measure of plant performance, they often exhibit different spatial patterns when compared to existing USA soil maps that are generated from limited soil sampling and landscape characteristics (i.e., topography) and mapped to discrete polygons that depict soil type (e.g., Soil Survey Geographic Database; SSURGO)^57^. YSZ effectively integrate the effects of soil formation factors, management, and plant-soil feedback on the heterogeneity of agricultural soils, arguably essential for the development of meaningful site-specific management zones. Their routine and expanded use will be important in the short- and longer term, and for individual producers and society at large, through for example informing precision N fertilization management that reduces farm costs and delivers improved water quality, and via more accurate and precise SOC stock quantification, integral to increasing carbon market payments to producers for their improved management practices, and for reducing greenhouse gas emissions associated with agricultural production.

In our study, we hypothesize that by observing and quantifying the long-term crops yields in intensively managed agricultural land through use of YSZ, we can stratify the in-field heterogeneity of soil conditions in terms of soil health attributes, including SOC, with differences that are resilient to various regional soil formation factors and management practices. Our hypothesis further suggests that the presence of yield stability and a particular level of yield within stable zones (i.e., low, medium, or high) points to differences in soil health attributes within a field that result from variations in the rates of soil formation and the accumulation of SOC due to topography, management, and plant-soil interaction effects. To investigate this, we address three questions: (1) What is the magnitude of the correlation of regional (between fields) soil formation factors on soil health and SOC relative to local (within fields) scale variability? (2) Do yield stability zones (YSZ) identify and characterize local scale soil health heterogeneity? And if so, what is the magnitude and direction of this relationship? (3) Does field management relate to the local scale variability of yield level? Our work runs parallel with Leuthold et al. 2023 (in review)^63^, who address the fractionation of soil organic matter (SOM) by YSZ in the same fields. By using long-term plant performance as an indicator of soil health through the use of YSZ, we aim to reduce the costs and complexities of sampling whilst enhancing the accuracy of assessments of soil characteristics and the evaluation of the spatial variability of SOC stocks. Answers to our questions will help to identify the drivers of the variance of soil health at small and large scales, both temporally and spatially, and can be used to help increase the adoption and accuracy of precision agricultural management and SOC stock evaluation.

## Methods

### Site description: climate and soil characteristics

**Figure 1:**
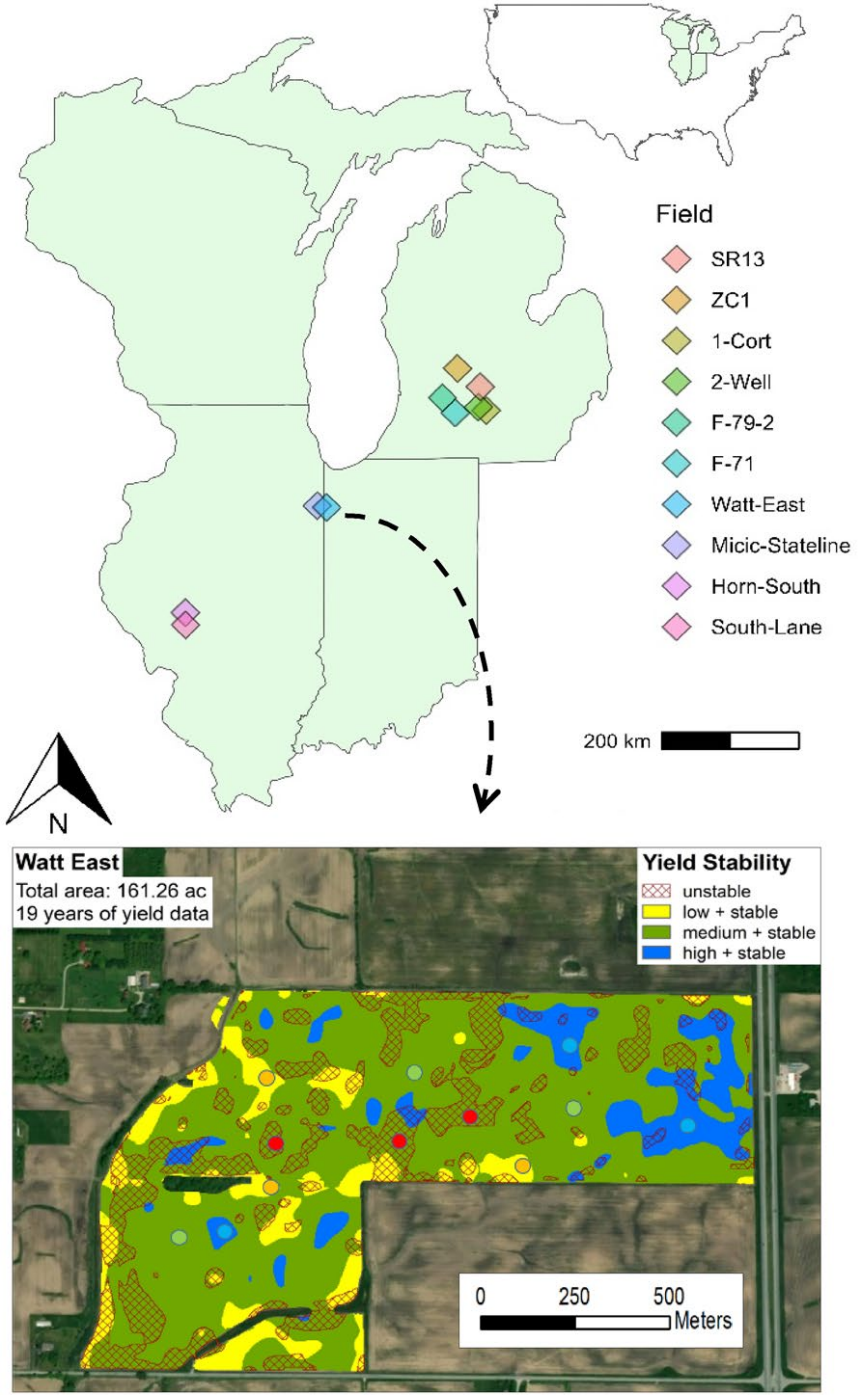
The field site locations in Michigan, Illinois, and Indiana, with an example field showing the three soil sampling locations (colored circles) randomly selected within each of the four levels of yield stability zones (YSZ); unstable (US), low and stable (LS), medium and stable (MS), and high and stable (HS), determined in each field. Map created by Ruben Ulbrich using ArcGis software 10.8.2. (www.esri.com).

Ten fields across eight commercial farms in Michigan, Illinois, and Indiana, covering an east–west gradient across the north-central Midwest region, were used in this study (Fig. 1). Mean annual precipitation (MAP) and temperature (MAT) ranges (Gridmet, 2000–2020, Table 1) in this area were 929–1045 mm and 9.1–12.1 °C, respectively^64^. Soil texture data were obtained from the spatial SSURGO dataset using a weighted depth for the sand, silt, and clay percentages from the dominant soil component of the map unit for each sample point^65^. The soils exhibit substantial variability, with the average clay content ranging from 9.3 to 30.4% across the fields.

**Table 1:** Field name, average precipitation (MAP), temperature (MAT), and soil texture.

State	Field	MAP (mm)	MAT (°C)	Clay (%)	Silt (%)	Sand (%)
Michigan	SR13	929	9.1	20.8	36.9	42.3
Michigan	ZC1	955	9.1	21.6	35.3	43
Michigan	1-Cort	938	9.3	15	29.6	55.4
Michigan	2-Well	949	9.3	12.6	25.6	61.8
Michigan	F-79–2	1024	9.9	9.3	23.4	67.3
Michigan	F-71	1045	10.1	10.6	26.9	62.6
Indiana	Watt-East	1042	10.1	30.4	57.8	11.7
Indiana	Micic-Stateline	1017	10.2	27.4	57.1	15.5
Illinois	Horn-South	1000	12.1	29	67.2	3.8
Illinois	South-Lane	1000	12.1	29.3	68.2	2.5

### Crop management

All fields were planted by the farmers to either corn or soybean in the year of soil sampling (2021). Management practices varied by farm and field and were not scientifically controlled for in our study; the ten fields had an equal split of tillage and no-tillage, with eight planted to cover crops, and all receiving split N applications, six with precision, prescription N side dress applications but with recommendations from different agronomic advisors (Table 2).

**Table 2:** Field location, name, area, crop rotation (2019–2021).

State	Field	Area (ha)	Crop rotation	Tillage	Corn N practice	Cover crop	Sample date Y/M/D
Michigan	SR13	38	Soybean/Wheat/Corn	Tillage	Prescription	Yes	2021/06/06
Michigan	ZC1	33	Soybean/Wheat/Corn	Tillage	Prescription	Yes	2021/06/07
Michigan	1-Cort	19	Wheat/Soybean/Corn	Tillage	Prescription	No	2021/06/06
Michigan	2-Well	1486	Soybean/Wheat/Corn	No-Till	Prescription	Yes	2021/06/06
Michigan	F-79_2	11	Soybean/Wheat/Corn	No-Till	Uniform	Yes	2021/06/05
Michigan	F-71	7	Soybean/Wheat/Corn	No-Till	Uniform	Yes	2021/06/05
Indiana	Watt-East	54	Soybean/Corn/Soybean	No-Till	Prescription	Yes	2021/07/07
Indiana	Micic-Stateline	69	Corn/Soybean/Corn	No-Till	Prescription	Yes	2021/07/07
Illinois	Horn-South	21	Wheat/Corn/Soybean	Tillage	Uniform	Yes	2021/07/07
Illinois	South-Lane	38	Wheat/Corn/Soybean	Tillage	Uniform	no	2021/07/30

Soil sampling date, and management practices.

### Yield stability zone determination

We determined yield stability zones (YSZ) in each field using high-resolution, gridded yield monitor data downloaded from harvesting machines as a semi-regular point shape file and interpolated to a two-meter resolution using ordinary kriging conducted using the arcpy library in python. The yield history of each field spanned a range of between 11 to 18 years, except for 1-Cort where 6 years of NDVI data were used as a proxy following the method of Basso et al. (2019)^44^. The determination of YSZ from yield monitors followed the methodology of Maestrini and Basso (2018, 2021)^33,57^. Briefly, we centered the yield value of each pixel by subtracting the field average for each year, then averaged the relative yield of each pixel across all years and calculated the yield level for each pixel. Low yields were stipulated as less than 10% from the field average, high yields greater than 10% from the field average, with medium yields occurring between these two values. Yield stability was then calculated from the standard deviation (SD) of each cells’ normalized yield over time. We calculated the SD of the relative yield for each field for each year, and then found the cell-by-cell average SD across all years. Stability for each cell was defined as unstable (US) when the SD was greater than + / − 15%, and stable otherwise. These thresholds of yield level combined with yield stability produce four stability zones, high and stable (HS), medium and stable (MS), low and stable (LS), and US.

### Soil sampling

Our sampling approach does not attempt to capture and characterize the entire soil variability in any one field, but does provide an even sample distribution by class of the yield stability zones (Table 3). Soil cores (0–30cm) were taken using a stainless-steel probe (MS Inc., American Falls, ID 83211; 5.01 cm internal diameter) and collected during the active crop growth period (June 5th to July 30th) in 2021 at three locations in each of the four YSZ (HS, MS, LS, UN) in each field (10) for a total of 120 cores. PVC core liners and caps were used to maintain soil integrity. Cores were split into 0–15 cm and 15–30 cm depth sections, generating 240 samples that were analyzed by a commercial laboratory (Woods End Laboratories LLC, Mt Vernon Maine 04352) commonly used by producers and stakeholders active in MRV development and the carbon marketplace to determine soil health metrics^50,66^.

**Table 3:** Sample distribution by yield stability zones across the ten fields in the US Mid-West.

Yield stability zone	Total area (ha)	Percent area (%)	Samples (0–30 cm)	Sample density (ha)
Unstable	83	18	30	6.8
Low stability	43	9	30	3.5
Medium stability	272	61	30	22.4
High stability	52	11	30	4.2
Total	449	100	120	9.2

A brief overview of the soil analytical tests is provided here, with more details provided in the Supplemental Information (SI; S1). To analyze our soils, we used the Solvita Nexus protocol, a test with a near 30-year commercial history. Samples were processed and analyzed for standard physical and chemical properties^23,67,68^, and for biological indicators including soil respiration^69^, potentially mineralized N (PMN), and Solvita labile amino nitrogen (SLAN)^70–72^. Water soluble organic carbon (WSOC) and water-soluble C:N ratios were as reported by Haney et al. (2012)^21^, and water stables aggregates (WSA) as determined by wet sieving according to Jemison et al. (2019)^73^. The combined suite of physical, chemical, and biological tests are detailed in Woods End Labs LLC, Soil Test Guidelines V2.1, 2023. The method for the integration of these metrics to develop a soil health score (SHS) and overall fertility score (OFS) is described below.

### Solvita Nexus soil health score (SHS)

The SHS is determined from six indicators (equally weighted, mg kg dry soil^-1^ unless otherwise noted; Eq. (1): CO_2_-burst, Solvita-color (SOL, log optical density), Solvita labile amino nitrogen (SLAN-N), water stable aggregates (WSA, volume %), water-soluble organic carbon (WSOC), and SOC^73^ as;

1 $$SHS = 10 x \left\{\frac{CO2x}{CO2d} + \frac{SOLx}{SOLd} +\frac{SLANx}{SLANd} +\frac{WSAx}{WSAd} +\frac{WSOCx}{WSOCd} +\frac{SOCx}{SOCd} \right\},$$

where x denotes the sample measurement and d is a maximum value scaling factor (units as above) of 250, 5.25, 400, 80, 400 and 3.5, respectively for CO_2_-burst, SOL, SLAN, WSA, WSOC, and SOC. The CO_2_-burst, also known as the CO_2_-flush^74–76^ (see SI S1). This equation results in a general SHS score of between 0 and 50. The general scores were localized using SSURGO sub-ordered types and an algorithm similar to the Soil Health Assessment Protocol and Evaluation tool^77^.

The overall fertility score (OFS), an index that combines SHS and ranking nutrients relative to an expected range, is then determined as:

2 $$OFS =\frac{NI}{2} + SHS$$

where NI is a nutrient index (%), i.e., the samples’ relative nutrient score determined by using the phosphorus and potassium analytical results from a Mehlich 1 extraction, and available-N (total water soluble-NO_3_-N + N-min, where N-min is estimated from the CO_2_-burst as modified^78–80^ relative to recommended values for the specific crop need^81^).

### Statistical analysis

We analyzed data at the regional scale, the local level, and with respect to field management practice to determine the relationship between YSZ with SHS, OFS, SOC, and component metrics of soil health. All analysis was performed in R^82^.

To assess the variability of soil health and SOC with regional scale and sub field scale soil formation factors, we created a correlation matrix and conducted a stepwise multiple linear regression (MLR) on those variables identified as statistically significant (P < 0.05) by the correlation analysis. We analyzed soil relationships for the 0–15 cm, 15–30 cm, and the combined 0–30 cm depth increments (n = 120), and present the 0–30 cm correlation analysis and MLR in the main results text for the local and regional variable analysis. We present the correlation and MLR analysis of all depth increments in SI (SI S2). We used the step package, a base R package to find the optimal model using the Akaike Information Criterion (AIC). We also determined the distribution of each of these variables and assessed their fit with assumptions of linearity, reliability of measurement, homoscedasticity, and normality^83^ (SI S2).

For regional soil formation factors, we evaluated the absolute values of SOC and SHS with the environmental variables MAP, MAT, % sand, % silt, and % clay, the topographic variables aspect, slope, and log of flow accumulation, and the latitude and longitude of each field. Flow accumulation is defined as the up-slope area that could contribute runoff at a point. The distribution of flow accumulation is log normal, so we log transformed this variable. Soil texture, climate, and digital elevation data were collected using the FedData package^84^. We collected elevation data from National Elevation Database at 1/3arc (10 m)^85^ and calculated the topographical variables aspect, slope, and flow accumulation using d8 algorithms from the whitebox package^86^.

We repeated the correlation and MLR analysis for local scale soil formation factors by analyzing the field relative variance of SOC and SHS with local soil formation factors. We normalized SOC and SHS response variables and soil texture values to the field with a z-score, subtracting the observed value by the field mean and dividing by the SD. We also analyzed the absolute values of topography. The correlation analysis included the components of the SHS to clearly show the interrelationship of these terms.

Then, using ANOVA, we evaluated the ability of YSZ to define statically different populations of field normalized SOC^63^, and SHS and its components, to assess the 95% confidence interval pair wise comparison (Tukey's honest significance test). We showed that the sample variance of each YSZ is approximately the same as with a Levene test (SI S2). We then conducted a post-hoc power analysis using the bootstrapped ANOVA from the YSZ sample population SOC to confirm that our sample design was appropriate to determine significant differences, and calculated the eta squared effect size to define the degree of difference between YSZ using the effectsize package^87^.

Finally, we assessed the degree of in-field heterogeneity of the absolute SOC and SHS as defined by the difference between the mean of the HS and LS zones in each field when compared to the categorical management practices (i.e., the presence or absence of tillage, cover crop, and variable rate nitrogen application). Note again that management practices were farmer not scientifically controlled in our study, and that the number of fields (n = 10) limited our statistical evaluation, but helped us to identify potential trends for further investigation. Reproducible scripts are available in the SI (S3).

## Results

To better evaluate the use of YSZ for identifying the spatial patterns of soil heterogeneity, soil health, and SOC, we divide this section into three. First, we present comparisons of the relationships between regional and local scale soil formation factors with soil health and SOC using correlation and multiple linear regression analysis. Second, across all fields we show the statistical differences of soil health, SOC, and other selected soil health metrics with YSZ categories. Third, we show the differences in soil health and SOC between the HS and LS zones across the differing field management practices.

### Soil formation factors and soil health

**Figure 2:**
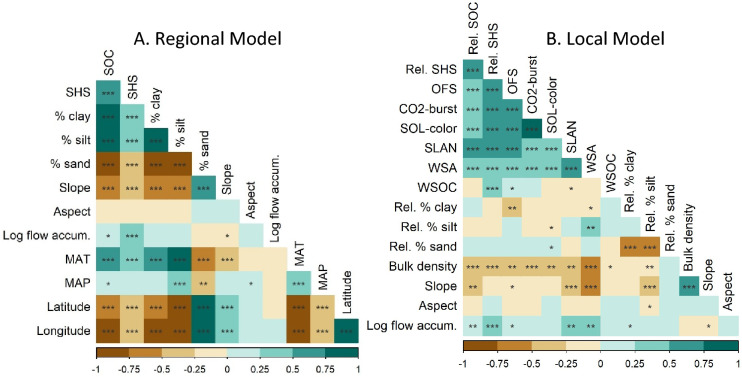
(**A**) Regional correlation the 0–30 cm depth soil organic carbon (SOC) and soil health score (SHS), soil texture (% clay, % silt, % sand), average topography (slope, aspect, and log flow accumulation (accum.), and long-term average annual temperature (MAT) and precipitation (MAP), and field location longitude and latitude. (**B**) Local correlation of relative (0–30 cm depth) soil organic carbon (SOC), soil health score (SHS), and overall fertility score (OFS) with the measured metrics of SHS (CO2-burst, Solvita (SOL) color (SOL-color), Solvita labile amino nitrogen (SLAN-N), water stable aggregates (WSA), water-soluble organic carbon (WSOC)), and soil texture, bulk density, and topography. Significance of the pairwise comparisons is denoted as ***<  = 0.001, **<  = 0.01, *<  = 0.05.

At regional scale, absolute values of SHS and SOC varied with soil texture, topography, and climate. The regional correlation analysis for the full sample depth (0-30cm) identified a high degree of co-linearity between the SOC, SHS, % clay, and % silt; the % clay of all fields correlated with SOC (R^2^ = 0.77, p = 2.0 × 10^–25^) and MAT (R^2^ = 0.56, p = 4.7 × 10^–11^), while MAT also correlated with SOC (R^2^ = 0.66, p = 4.8 × 10^–16^) (Fig. 2, tabluated values are presented in SI S3 Tables S2 and S3). These relationships are geographically correlated, with the average SOC, SHS, % clay, % silt, and MAT all decreasing in value as the sampling transect heads to the northeast. For the regional model we determined an adjusted R^2^ of 0.70 (p = 2.2 × 10^–16^) and 0.27 (p = 6.5 × 10^–9^) for SOC and SHS, respectively (Table 4). The % clay (p = 1.2 × 10^–7^), % sand (p = 6.5 × 10^–4^), and MAT (p = 3.3 × 10^–8^) being significant factors correlated with SOC.

**Table 4:** Stepwise multiline regression model and statistics for regional and local covariates for soil organic carbon (SOC) and the Soil health Score (SHS) for the full sampled depth (0–30 cm).

Scale	Model	R2 adj	P
Regional	SOC = 0.12 (% clay) + 0.03 (% sand) − 0.39 (MAT) − 6.06	0.70	2.2 × 10^−16^
Local	Rel. SOC = 0.37 (Rel. % clay) − 0.19 (Rel. % sand) − 0.12 (slope) + 0.21 (log flow accum.) + 0.04	0.17	3.9 × 10^−5^
Regional	SHS = 0.69 (% clay) + 0.21 (% sand)- + 1.9 (MAT) − 20.9	0.27	6.5 × 10^−9^
Local	Rel. SHS = − 0.39 (Rel. % silt) − 0.29 (Rel. % sand) + 0.36 (log flow accum.) − 0.34	0.17	1.7 × 10^−5^

At the local scale, the relative variability of SOC and SHS within the fields correlated with z-scored soil texture and topographic indices, but with much weaker relationships than with predictors and the absolute values of SHS and SOS across the regional transect. Topography (slope and log flow accumulation), field relative % clay, and % bulk density all correlated significantly with SOC. The SHS was also correlated with flow accumulation, bulk density, and the log of the flow accumulation. The SHS did not vary significantly with slope. The scaled SOC and SHS, and the absolute components of the SHS are all strongly colinear (Fig. 2). The MLR model of the 0–30 cm soil depth provided limited explanation of the field relative SOC and SHS, with an adjusted R^2^ of 0.17 (p = 3.9 × 10^–5^) and 0.17 (p = 1.7 × 10^–5^), respectively.

### Yield stability zones and soil health

**Figure 3:**
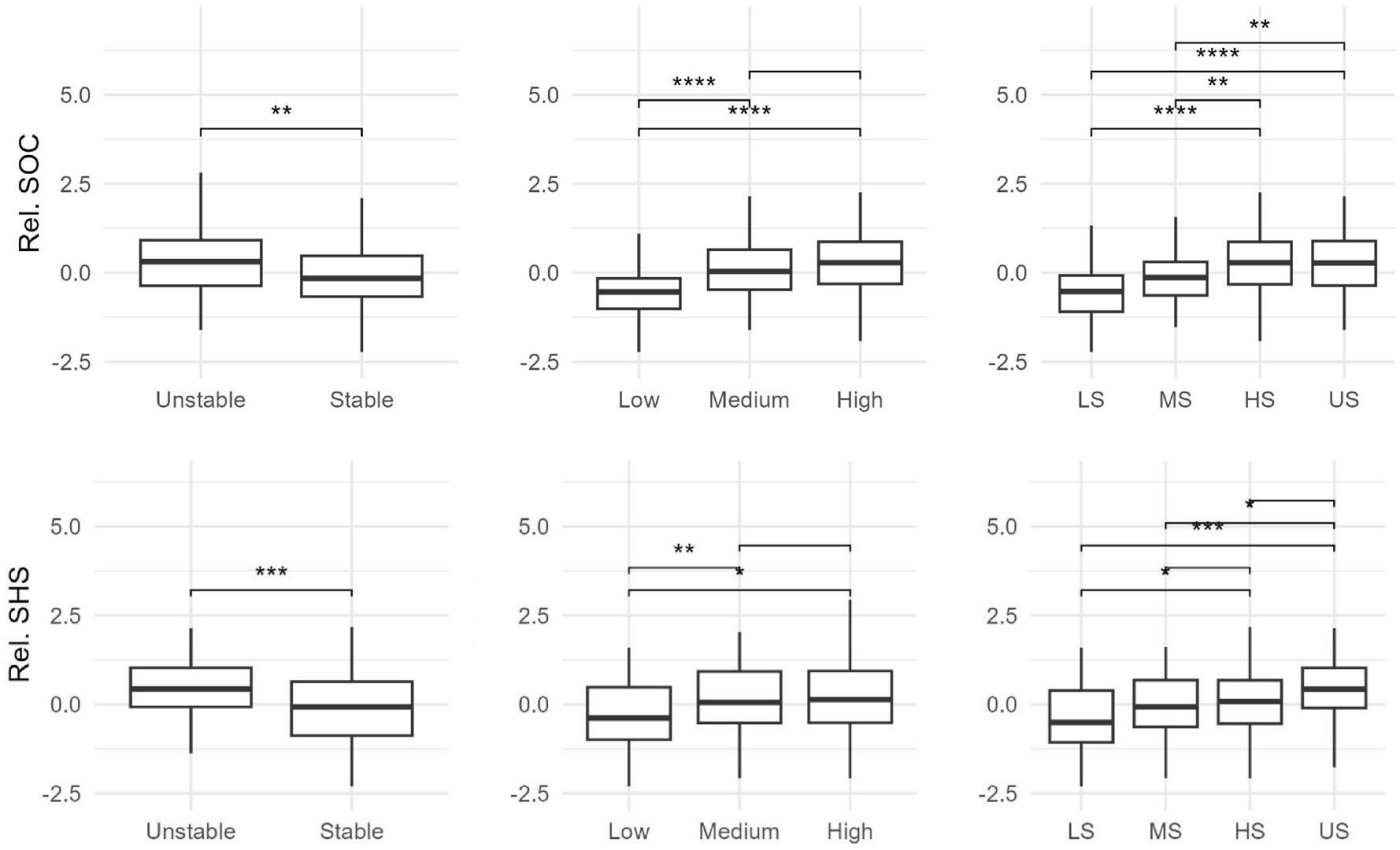
Separation of field-relative (Rel.) soil organic carbon (SOC) and soil health score (SHS) by yield stability (left; unstable and stable), yield level (center; low, medium, and high), and yield stability zone (right; low and stable [LS], medium and stable [MS], high and stable [HS] and unstable [US]). Significance of the pairwise comparisons is denoted as ****<  = 1 × 10^–04^, ***<  = 0.001, **<  = 0.01, *<  = 0.05.

Across all fields, the field-relative SOC and SHS values for the 0–30 cm soil depth varied significantly between yield stability, yield level, and YSZ (Fig. 3). In particular, the unstable zone contains significantly more SOC and has a higher SHS compared to the stable zones. For yield level, the relative SOC and SHS in the low yield zone are significantly different when compared to the medium and high yield zones (see Fig. 5 and SI Fig. S11 for 95% CIs). A power analysis of the SOC difference between the 30 samples taken in each YSZ zone across the ten fields simulated a power value of 94.7% over 10,000 bootstrapped iterations, with an effect size of 0.09, 0.18, and 0.21 for the 0–15 cm, 15–30 cm, and combined 0–30 sample depths, respectively. This indicated a small effect for the surface soil and a medium effect for the lower depth increment and the full sample depth. When comparing YSZ across all fields, the relative SOC and SHS in the US zone does not differ significantly from the HS zone but does from the LS and MS zones. The average of the relative mean SOC of the US zone is greater than the average of the LS and MS zones by 0.81 and 0.43 SDs, respectively. Similarly, the average of the relative mean SOC of the HS zone is greater than the average of the LS and MS zones by 0.89 and 0.51 SDs, respectively. The average absolute difference of the SOC concentration between the HS and LS zones is 0.42% SOC, representing a 37.6% difference (See SI Table S1 for field level YSZ absolute mean and SD values).

**Figure 4:**
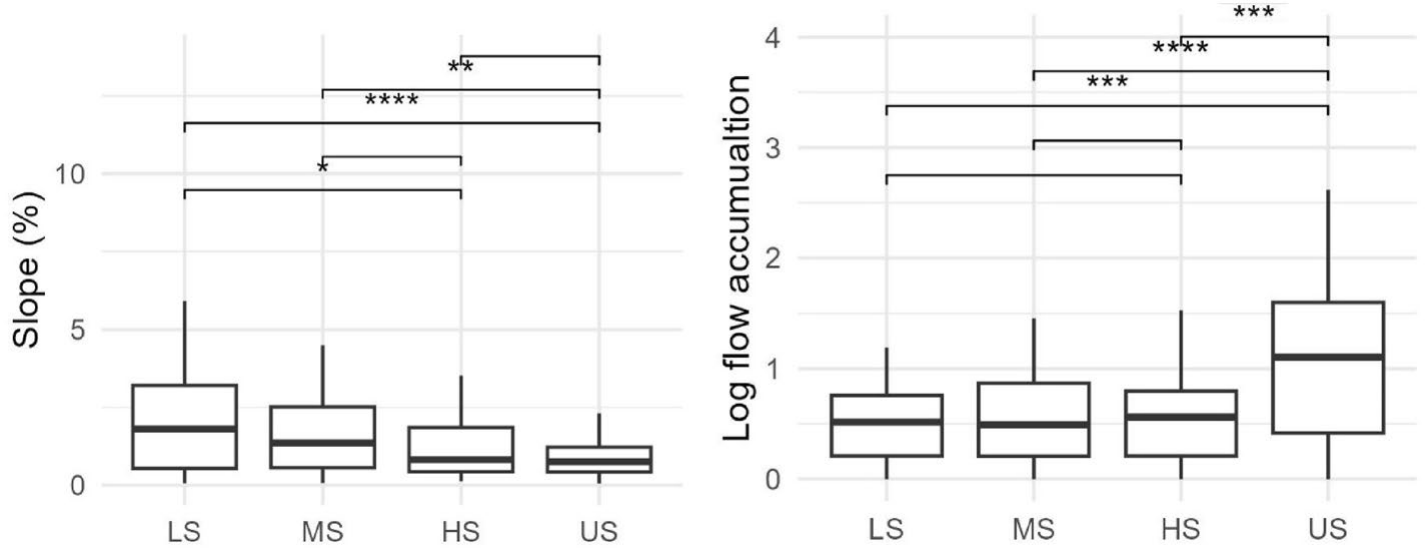
The distribution of yield stability zone (YSZ) by percent slope (left) and the log of the flow accumulation (right). See text and Fig. 4 for YSZ terms. Significance of the pairwise comparisons is denoted as ****<  = 1 × 10–04, ***<  = 0.001, **<  = 0.01, *<  = 0.05.

The topographic indices, extracted from spatial data at the sample locations, were significantly different between YSZ. The HS and US zones predominantly occurred on low slopes with the slope average for a sample point of 1.3% and 0.9%, respectively across all fields. The HS was statistically different from LS, and the US was statistically different from LS and MS with average slopes at the sample locations of 2.3% and 1.7%, respectively. The US zones were more prevalent in areas with high flow accumulation when compared to all the stable zones (Fig. 4), with average log flow accumulations of 1.15, 0.59, 0.61, and 0.56 for the US, HS, MS, and LS, respectively. The aspect and field relative soil texture showed no significant differences between YSZ at the field scale (see SI Fig. S12 for 95% CIs).

**Figure 5:**
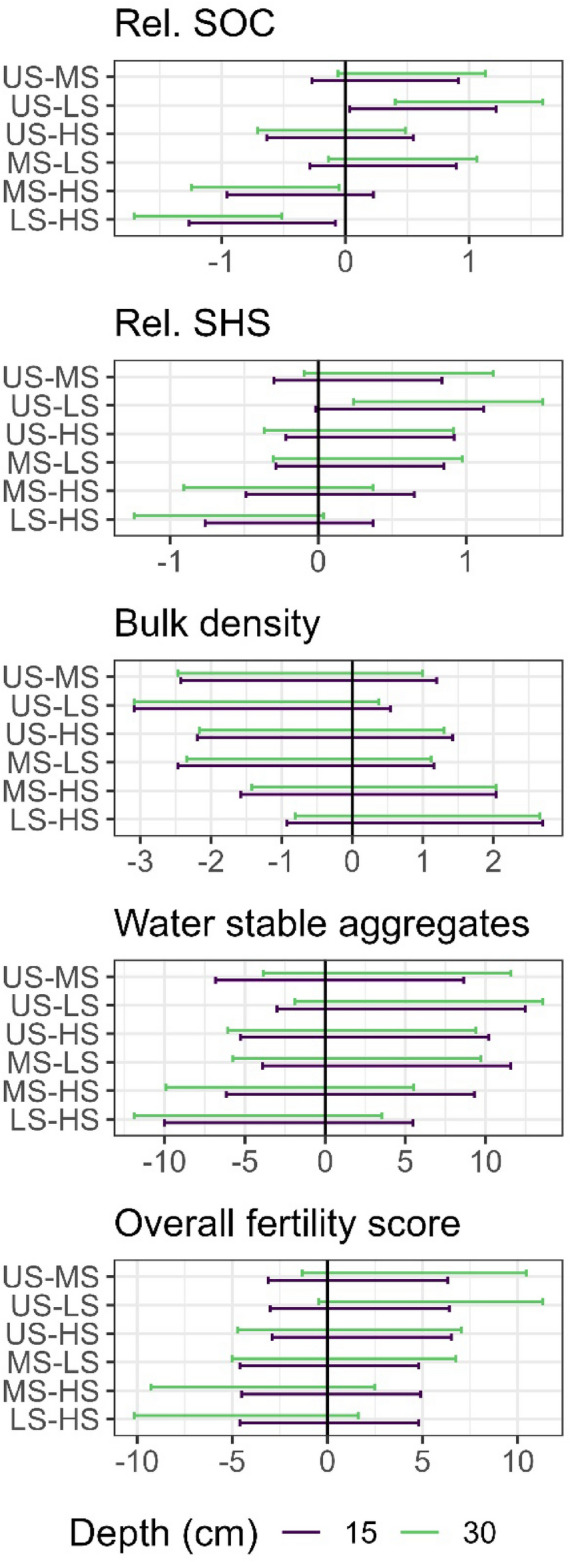
The 95% confidence interval of statistical variance between stability zones (YSZ) pairs for field relative soil organic carbon (SOC), and soil health score (SHS), bulk density, water stable aggregates, and overall fertility score at 0-15cm (purple) and 15-30cm (green) soil depths. See text and Fig. 4, For YSZ terms. The YSZ are defined as low and stable (LS), Medium an stable (MS), High and stable (HS) and Unstable (US).

Differences in soil physical (bulk density and water stable aggregates) and chemical (OFS) measures of soil health, commonly associated with SOC, followed consistent trends with YSZ, that increased in difference with increasing soil depth increments (Fig. 5). The significance of the difference between YSZ for both relative SOC and SHS also increased consistently with the soil depth increment. The 95% confidence interval of the difference between HS-LS and US-LS increased in their degree of difference for the 15–30 cm depth compared to the 0–15 cm depth. While not statistically significant, quantitative measures of soil quality; bulk density, water stable aggregates (WSA) and overall soil fertility (OFS), mirrored the trends in the YSZ separation of in-field heterogeneity. The components of SHS capture these trends with SLAN significantly different between US-LS, US-MS, and HS-LS (see SI Fig. S13 for 95% CIs).

### Yield stability zones and management

**Figure 6:**
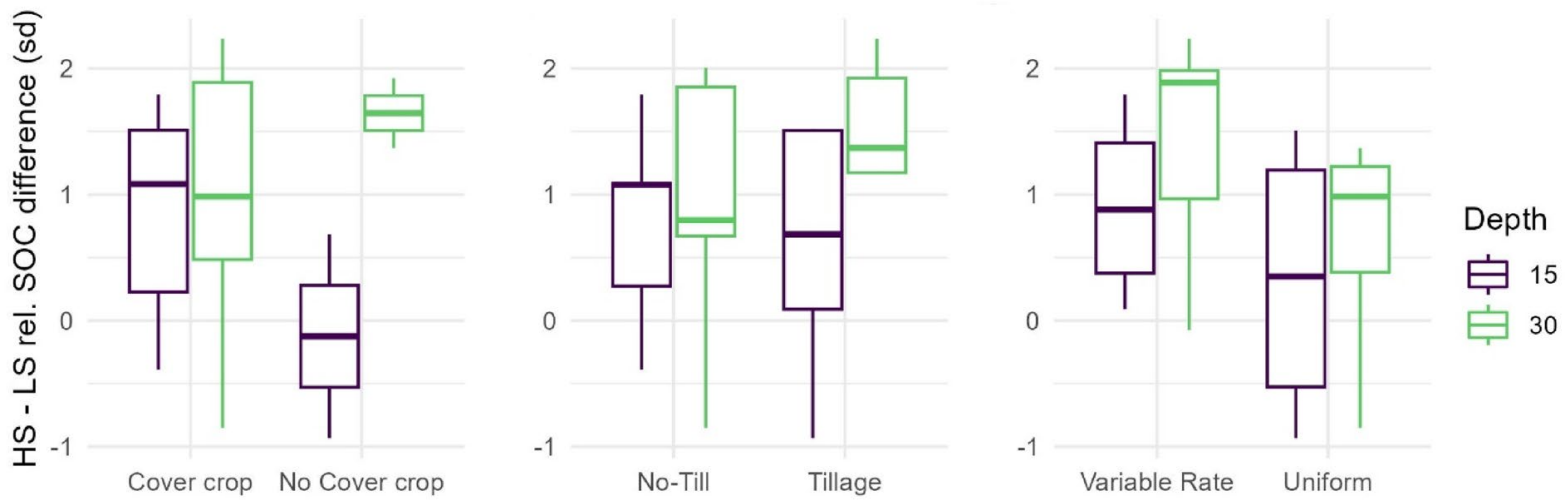
Effect of field management practice (cover crop presence or absence, no-tillage or tillage, and nitrogen rate (uniform or variable) on the average difference in normalized SOC between HS and LS zones for soil depths 0-15cm (15) and 15-30cm (30).

Yield stability zones identified persistent differences in SOC across fields between conventional (e.g., tillage) and regenerative (e.g., no-till) management practices (Fig. 6). The difference in field relative SOC between HS and LS was positive (i.e., > 0) for the majority of sample points because SOC in the HS zones was higher than in LS soils. The practice of no-till showed an increased difference in the normalized SOC between the HS and LS zones at the 0–15 cm depth (0.39 SD) but showed an decreased difference at the 15–30 cm depth (− 0.57 SD) (Fig. 6). The use of cover crops similarly showed an increased difference in relative SOC in the surface soil (1.21 SD) and a decreased difference in the subsurface soil (0.62 SD). The use of variable-rate prescription N applications showed an increased difference in relative SOC between HS and LS zones by 0.53 and 0.90 SD for the 0–15 cm and 15–30 cm soil depth increments, respectively.

## Discussion

In this study, we have evaluated the hypothesis that yield stability zones (YSZ) can be used to identify distinct regions within a field of SOC and soil health, due to the integrated effects of topography, management, and plant-soil feedback.

### Soil formation factors and soil health

At a regional scale, we found that the average field values of SOC and SHS were predicted by soil texture and mean average temperature (MAT), with adj. R^2^ of 70% and 31%, respectively. Long-term soil formation factors of climate, time, and parent material strongly regulate the weathering of rocks into fine soil particles that promote SOC storage as mineral associated organic matter and aggregation formation. These effects challenge the direct measurements of soil health or SOC changes with management across a region because the measurement signal is small relative to the spatial noise. We reaffirm the need to use locally scaled measures of soil health to evaluate and inform management^88–90^ and to guide SOC sampling for MRV applications^91^.

At the subfield scale, the relative variations in SOC and SHS were also explained by soil texture and topographic factors, but the coefficients of determination (R^2^) were much lower; 10% and 12% respectively. Topography provides first and second order drivers of SOC accumulation. Erosion transports SOC down slope directly, then nutrients and water flow downhill providing the resources for greater plant growth^37,92,93^, but these mechanisms are weak relative to the spatial heterogeneity of soil health. Historically, farming practices have resulted in soil degradation due to the mining of soil nutrients, depletion of SOC, soil erosion, and soil compaction^94^. As a result, a focus on local, management driven changes in soil chemistry and biology has diverted attention on the effect of soil formation factors on soil health in the literature^7^. To better integrate all drivers of soil health, we need an approach that better encompasses these geomorphological forces and plant-soil interactions to help inform improved management options. Here, we demonstrate that YSZ can capture these integrated patterns in space and time by identifying statistically significant stratification of the field-relative SOC and soil health across various soil types and management practices.

### Yield stability zones and soil health

Unstable zones exhibit higher levels of SOC and SHS compared to all the stable zones, with these differences related to topographic factors^63^. Unstable zones can intermittently produce high yields similar to the high and stable zones, but at other times produce low yields due to seasonal weather conditions. The expectation that increasing SOC and SHS increase yield stability^95^ is still valid, because overall, unstable zones are typically high yielding rather than low yielding. Our findings suggest that topography outweighs localized plant-soil interactions^37^. We found an increase in SOC and SHS with increasing average yield, an intuitive result corroborated by others^41,95,96^. The difference in SHS and SOC between YSZ, yield stability, and yield level groupings was largely driven by LS zones which are found on steeper slopes. The significantly higher BD in LS zones compared to HS zones, suggests potential compaction or a reduced topsoil depth due to erosion^93^. Overall, and particularly in terms of increased water and nutrient holding capacity, our findings indicate that YSZ can effectively integrate the relationships between soil formation, SOC accumulation, soil health, and yield potential.

Specifically, the relationship between YSZ and SOC distribution could be used to reduce the sampling error in estimating SOC stock accrual or depletion over time. Many carbon market MRV protocols require sampling across multiple time points and recommend or require stratification, but do not provide adequate quantitaive methods^91^. Meta analysis of land use change impacts on SOC due to for example, the adoption of no tillage or the use of cover crops, show a typical increase in SOC of 10%^97^. In our study, we found that the average difference in SOC concentration between the LS and HS zones within a single growing season across ten fields to be 37.6%, almost four times as great as the changes due to the adoption of regenerative management practices. This heterogeneity has major implications for protocols that require consecutive sampling to determine SOC stock change, and strongly reiterates the need for resampling at the exact prior location, an action which practically is very difficult to achieve. Notithstanding, when sampling across time, the use of YSZ provides an important means of stratifying SOC samples, and improving the accuracy of SOC stock change evaluation over time.

### Yield stability zones and management

Across all fields and management practices the difference in relative SOC between HS and LS remained positive. The use of cover crops or no-till resulted in a reduced difference in SOC between the HS and LS zones in the 15–30 cm soil depth, but an increased difference in the 0–15 cm depth, when compared to their conventional alternatives. Cover crops and no-till practices have been associated with increased yield stability^98^ and may reduce the difference in SOC at depth by reducing soil and carbon transport down hill^99,100^, thus further implying that LS zones are developed through a combination of soil loss and a feedback with reduced primary production. Our data suggest that the presence of tillage or more bare soil (i.e., the absence of cover crops) may result in a more consistent surface soil layer depth across the YSZ within a field. However, more samples over a wider geographic area are needed to investigate the formation of LS zones, and to help further seperate erosion from other plant-soil interactions that can limit SOC accumulation. Variable rate N prescriptions increased the difference in SOC between HS and LS zones for both depth intervals. Nitrogen fertilization is well known as a first order control on crop biomass and yield. While YSZ can delineate site specific management zones to construct fertilization prescriptions^101^, we expect yields to respond to those prescriptions. If all the N fertilizer applied was used by the plants, the yield capacity of the soil would still be captured by the YSZ, because of water and other nutrient limitations. However, fertilizer prescriptions are imperfect, and ensuring high nitrogen use efficiency is difficult^102^; even small yield changes near optimum N application impact YSZ.

### Methodological limitations

The generalization and reproducibility of soil health measurement due to changes in management is debated. Comparisons between the various tests, including Alabama and Cornell^24^ and Cornell and Haney^103^ show contrasting results for overall soil health scores. Variations in regionally unique soil properties may influence these scores more than management-related differences^24,103^. This lack of sensitivity is due in part to the high spatial and temporal variability of soil properties. When aggregating unique managements and sites, the mean of soil health metrics has shown distinct differences and strong correlation with yield^10^. Without standardized segmentation and identification of the management population mean and variance, the confounding effects of soil properties on soil health scores limit their intercomparison^98^. Based on this understanding, we addressed the spatial variability by evaluating regional factors of soil health and field-relative spatial patterns. We identified YSZ as a meaningful spatial segmentation strategy but have not determined a specific sample number of samples needed to identify the mean soil health score or SOC value per YSZ in a specific field^104^.

Soil health is also likely to change over time both seasonally and over decades. While here we are comparing the spatial distribution of soil health and SOC to the long-term spatial pattern of yield, single time point sampling of soil health may introduce variance associated with short-term dynamics. Soil health metrics change with moisture and nutrient conditions through a growing season^105^. Further analysis of the total duration of a management and in-season variability of soil health and SOC are needed. Given these limitations, our approach seeks to relate soil health with the spatial patterns of long-term crop yield to help define the patterns of soil health.

### Future work and outlook

Our YSZ management relationships are based on a relatively small data set, and further study across a wider range of soil types and climate conditions is ongoing in the Basso Lab at Michigan State University. Investigating and adapting YSZ to wider geographic and climatological regions is important future work to better deliniate the spatial variation of SHS and SOC to help increase yield, reduce pollution, and store more carbon in the soil. Results from the gentle topography of the USA Midwest may not transfer directly to other areas. Landscapes with greater slopes where lateral carbon fluxes, soil erosion, and colloidal transport are more significant may confound the relationship between SOC and YSZ. Similarly, arid environments under rain-fed conditions, where water is a more important limiting resource, may also impact the magnitude of YSZ differences^106^. In these scenarios, plant performance can still identify topographic effects, but regional specific YSZ differences observed in this study may not hold. Ongoing climate change will further complicate the use of YSZ for managing SOC and restoring degraded soils. Increased soil erosion, changes in precipitation patterns, and rising temperatures all can alter yield level and yield stability patterns, challenging the assumptions of the dynamic equilibrium underlying YSZ approaches^107,108^. Adaptive spatially variable agronomic management strategies will be crucial to mitigate these impacts, protect soil resources, and account for heterogeneity in yield level and stability under dynamic climate conditions. Understanding that the integrated effects of topography, plant-soil interactions, and management history connect yield, YSZ, and soil health brings us closer to overcoming these challenges.

## Conclusion

Yield stability zones (YSZ) successfully identify areas of a field with statistically distinct, relative soil organic carbon (SOC) and relative soil health across different soil types and management practices. Trends in soil characteristics within YSZ indicate that LS zones tend to have shallower or more compacted soils, higher bulk densities and are located on steeper slopes, while unstable (US) zones exhibit higher SOC levels associated with flow accumulation and top soil accumulation from erosion processes. These findings suggest that YSZ can identify the feedback relationships between soil formation, SOC accumulation, soil health, and yield potential, particularly in terms of increased water and nutrient holding capacity. Yield stability maps maps provide valuable insights into soil health based on the long-term, topographic, and biological drivers of soil formation which are then reflected in spatial and temporal variation of crop yield and SOC accumulation.

### Supplementary Information

Supplementary Information.

## Data Availability

The datasets used during the current study are available from the corresponding author on reasonable request.

## Abbreviations

LS: Low and stable YSZ
MAP: Mean annual precipitation
MAT: Mean annual temperature
MRV: Measurement, reporting, and verification
MS: Medium and stable YSZ
N: Nitrogen
HS: High and stable YSZ
OFS: Overall fertility score
MLR: Multiple linear regression
SD: Standard deviation
SHS: Soil health score
SLAN-N: Solvita labile amino nitrogen
SOC: Soil organic carbon
SOL: Solvita-color
SSURGO: Soil survey geographic database
US: Unstable YSZ
WSA: Water stable aggregates-soluble organic carbon
WSOC: Water-soluble organic carbon
YSZ: Yield stability zone
