# Supplementary material for: Spatial patterns of historical crop yields reveal soil health attributes in US Midwest fields
Source: Sci Rep. 2024 Jan 3;14:465. doi: 10.1038/s41598-024-51155-y (PMC10764739; doi:10.1038/s41598-024-51155-y)
Supplement: Supplementary file 1 — Supplementary Information. [file 41598_2024_51155_MOESM1_ESM.docx]

# Supplemental information

## S1. Methods: Soil processing and analysis

Samples were placed in open drying dishes, dried to < 2% moisture over 24 hours, then sub-sampled for total carbon (TC) and soil health indices. Prior to TC analysis, soil was hammermilled and sieved (2 mm, Agvise Stainless Steel Grinder, Northwood ND). Prior to soil health analysis, soil was placed on a table covered with kraft paper, crushed (4 kg wooden rolling pin) then sieved (< 2 mm; ^1^). For field bulk density, split cores (0 – 15 cm and 15 – 30 cm) were dehumidified, weighed, broken up, crushed, and sieved (< 2 mm), with the > 2mm fraction (stones and gravel) weighed separately. Final bulk density was corrected for using two methods; subsampling to determine residual water after 105°C convection oven drying and determining the weight contribution from > 2mm fraction expressed as an actual soil loss of volume^1^.

For TC, 200-400 mg soil was weighed into a refractory porcelain boat and analyzed using a TOC analyzer (Shimadzu with SSM-5000A solid combustion unit, oven at 900°C, O_2_ carrier gas at 500 mL min^-1^, limit of detection = 0.1.mg as C, limit of quantification = 1.0 mg). Glucose and sodium carbonate standards were used with a linear 3-point calibration and linear detection of 1 – 50 mg C. Sample size was adjusted, and analysis repeated if response was outside this range. TOC was determined from the difference between the measured total carbon (TC) and total inorganic carbon (TIC). Results were corrected to an oven-dry moisture basis.

Additional processing and analysis of soil sub-samples for soil health indices are outlined below and along with TOC used to determine an overall soil health score (SHS).

*Water-soluble organic carbon (WSOC)*: A 1:10 soil:water extract (4g air-dried, <2 mm soil shaken for 10 minutes in 40 ml deionized water) was centrifuged, filtered (Whatman #2), had inorganic carbonates removed (H_3_PO_4_ to pH <3), and was analyzed in 100ul aliquots in a combustion oven (850°C) fitted with a O_2_ stream in a catalytic column with non-dispersive infra-red (NDIR) detection of CO_2_.

*Solvita labile amino nitrogen (SLAN)*: Dried and sieved (< 2mm) soil samples (4 cm^3^) were incubated (sealed jar, 24 hours, 20°C) in NaOH (20 ml, 2 N) with a NH_3_-trapping gel probe. After incubation, probe color was determined by colorimetry by comparing with two filtered wavelengths (640 nm and 470 nm) previously calibrated to NH_3_–N amount absorbed by thin-layer boric-acid gel (0 – 2 mg N range). Results are reported as SLAN–N mg kg dry soil^-1 2^.

*Solvita CO2-burst*: Dehumidified soil was adjusted to 50 % water-filled pore-space (WFPS) by drying, sieving, and weighing to nearest 0.1 g in a certified scoop (30 cm^3^). The bulk density mass was converted to absolute volume (soil weight / 2.65 cm^3^ g^-1^ particle density), subtracted from the total soil volume to determine available pore volume, then moistened to 50% of this volume by gently pipetting DI water on top, allowing downward percolation that marked the test start. After incubation (24 hours, 20°C) CO_2_ absorption was measured using the Solvita thin layer gel technique^3^, where the color result is quantified using colorimetry with filters for 550 nm and 470 nm from which CO_2_ % is derived by calibration equation with standard gases^4^. As a calibration check, 10% samples are checked against an IR analysis device (IRTH®, Woods End Laboratories) using the protocol of ^5^ .

*Water stable aggregates (WSA)*: The volumetric aggregate stability test (VAST) was used. The dried soil (< 2mm) was wetted on a sieve (35-mesh) using room temperature deionized water for 30 seconds to initiate disaggregation. After aggregate destruction the process was repeated and the sand fraction (> 0.5 mm) removed, with the resulting macro aggregates (0.5 - 2.0 mm) compared against a reference blank (pure sand pre-sieved to 0.5 -2.0 mm)^6^.

*Nutrient Index (NI):* the NI is a nutrient index (%), i.e., the samples’ relative nutrient score determined by using the phosphorus and potassium analytical results from a Mehlich-1 extraction, and available-N (total water soluble-NO_3_-N + N-min, where N-min is estimated from the CO_2_-burs. The calculation is as follows.

factor a (N) b (P) c (K)

Total NI = $\surd$ $\{ (Avail-N)/(N rate)+(Ext P-m)/(P rate)+(Ext K-m)/(K rate) \}$÷ 2

Where avail-N = NO3-N + N_min_ , *rate* is tabular nutrient requirement from SNAP for expected yield in bu/a, *Ext* is level extracted with Mehlich1; *m* is maintenance level set for each region. The overall index is capped at 100 ( $if NI >100 then NI=100)$. Simplified,

$$NI=\frac{\sqrt{a+b+c}}{2}$$

Or full version:

$$NI=\frac{\sqrt{(Avail-N)/(N rate)+(Ext P-m)/(P rate)+(Ext K-m)/(K rate)}}{2}$$

## S2. Statistical assumptions and model details

### Multilinear regression of SOC Regional stepwise model.

**Full depth 0-30cm SOC model**

Start: AIC=-228.25

soc ~ claytotal.r + sandtotal.r + tavg + pr

Df Sum of Sq RSS AIC

- pr 1 0.1230 16.602 -229.35

<none> 16.479 -228.25

- sandtotal.r 1 1.5644 18.044 -219.36

- claytotal.r 1 4.4255 20.905 -201.70

- tavg 1 4.9848 21.464 -198.53

Step: AIC=-229.35

soc ~ claytotal.r + sandtotal.r + tavg

Df Sum of Sq RSS AIC

<none> 16.602 -229.35

- sandtotal.r 1 1.6082 18.210 -220.26

- claytotal.r 1 4.5752 21.177 -202.15

- tavg 1 4.9276 21.530 -200.17

Call:

lm(formula = soc ~ claytotal.r + sandtotal.r + tavg, data = (farms_points_sf_15_all %>%

group_by(Rep, Farm, Stability) %>% summarise_all(mean) %>%

ungroup %>% dplyr::select(soc, claytotal.r, sandtotal.r,

tavg, pr)))

Residuals:

Min 1Q Median 3Q Max

-0.84515 -0.25247 -0.01423 0.20974 1.18481

Coefficients:

Estimate Std. Error t value Pr(>|t|)

(Intercept) -6.060210 1.307796 -4.634 9.46e-06 ***

claytotal.r 0.118391 0.020940 5.654 1.14e-07 ***

sandtotal.r 0.027331 0.008153 3.352 0.00108 **

tavg 0.390571 0.066564 5.868 4.27e-08 ***

---

Signif. codes:

0 ‘***’ 0.001 ‘**’ 0.01 ‘*’ 0.05 ‘.’ 0.1 ‘ ’ 1

Residual standard error: 0.3783 on 116 degrees of freedom

Multiple R-squared: 0.704, Adjusted R-squared: 0.6964

F-statistic: 91.98 on 3 and 116 DF, p-value: < 2.2e-16

**0-15cm depth SOC model**

Call:

lm(formula = soc ~ sandtotal.r + tavg, data = (farms_points_sf_15_all %>%

filter(Depth_cm == 30) %>% dplyr::select(soc, claytotal.r,

sandtotal.r, tavg, pr)))

Residuals:

Min 1Q Median 3Q Max

-1.0897 -0.2924 -0.0320 0.2668 1.9321

Coefficients:

Estimate Std. Error t value Pr(>|t|)

(Intercept) -0.608960 0.592070 -1.029 0.306

sandtotal.r -0.012069 0.001987 -6.072 1.61e-08 ***

tavg 0.228109 0.053262 4.283 3.80e-05 ***

---

Signif. codes:

0 ‘***’ 0.001 ‘**’ 0.01 ‘*’ 0.05 ‘.’ 0.1 ‘ ’ 1

Residual standard error: 0.4802 on 117 degrees of freedom

Multiple R-squared: 0.5476, Adjusted R-squared: 0.5399

F-statistic: 70.81 on 2 and 117 DF, p-value: < 2.2e-16

**15-30cm depth SOC model**

Call:

lm(formula = soc ~ sandtotal.r + tavg, data = (farms_points_sf_15_all %>%

filter(Depth_cm == 30) %>% dplyr::select(soc, claytotal.r,

sandtotal.r, tavg, pr)))

Residuals:

Min 1Q Median 3Q Max

-1.0897 -0.2924 -0.0320 0.2668 1.9321

Coefficients:

Estimate Std. Error t value Pr(>|t|)

(Intercept) -0.608960 0.592070 -1.029 0.306

sandtotal.r -0.012069 0.001987 -6.072 1.61e-08 ***

tavg 0.228109 0.053262 4.283 3.80e-05 ***

---

Signif. codes:

0 ‘***’ 0.001 ‘**’ 0.01 ‘*’ 0.05 ‘.’ 0.1 ‘ ’ 1

Residual standard error: 0.4802 on 117 degrees of freedom

Multiple R-squared: 0.5476, Adjusted R-squared: 0.5399

F-statistic: 70.81 on 2 and 117 DF, p-value: < 2.2e-16

Multilinear regression of SOC local stepwise model.

**Full depth 0-30cm SOC model**

Start: AIC=282.61

SHS ~ claytotal.r + sandtotal.r + tavg + pr

Df Sum of Sq RSS AIC

- pr 1 1.808 1165.4 280.80

<none> 1163.5 282.61

- sandtotal.r 1 91.469 1255.0 289.69

- tavg 1 117.410 1281.0 292.14

- claytotal.r 1 153.747 1317.3 295.50

Step: AIC=280.79

SHS ~ claytotal.r + sandtotal.r + tavg

Df Sum of Sq RSS AIC

<none> 1165.4 280.80

- sandtotal.r 1 92.843 1258.2 287.99

- tavg 1 118.213 1283.6 290.39

- claytotal.r 1 157.476 1322.8 294.00

Call:

lm(formula = SHS ~ claytotal.r + sandtotal.r + tavg, data = (farms_points_sf_15_all %>%

group_by(Rep, Farm, Stability) %>% summarise_all(mean) %>%

ungroup %>% dplyr::select(SHS, claytotal.r, sandtotal.r,

tavg, pr)))

Residuals:

Min 1Q Median 3Q Max

-10.3193 -1.8897 0.3421 2.3411 8.5649

Coefficients:

Estimate Std. Error t value Pr(>|t|)

(Intercept) -20.87262 10.95683 -1.905 0.059258 .

claytotal.r 0.69458 0.17543 3.959 0.000130 ***

sandtotal.r 0.20766 0.06831 3.040 0.002925 **

tavg 1.91300 0.55768 3.430 0.000836 ***

---

Signif. codes:

0 ‘***’ 0.001 ‘**’ 0.01 ‘*’ 0.05 ‘.’ 0.1 ‘ ’ 1

Residual standard error: 3.17 on 116 degrees of freedom

Multiple R-squared: 0.2967, Adjusted R-squared: 0.2785

F-statistic: 16.32 on 3 and 116 DF, p-value: 6.524e-09

**0-15cm depth SOC model**

Call:

lm(formula = soc ~ sandtotal.r + tavg, data = (farms_points_sf_15_all %>%

filter(Depth_cm == 30) %>% dplyr::select(soc, claytotal.r,

sandtotal.r, tavg, pr)))

Residuals:

Min 1Q Median 3Q Max

-1.0897 -0.2924 -0.0320 0.2668 1.9321

Coefficients:

Estimate Std. Error t value Pr(>|t|)

(Intercept) -0.608960 0.592070 -1.029 0.306

sandtotal.r -0.012069 0.001987 -6.072 1.61e-08 ***

tavg 0.228109 0.053262 4.283 3.80e-05 ***

---

Signif. codes:

0 ‘***’ 0.001 ‘**’ 0.01 ‘*’ 0.05 ‘.’ 0.1 ‘ ’ 1

Residual standard error: 0.4802 on 117 degrees of freedom

Multiple R-squared: 0.5476, Adjusted R-squared: 0.5399

F-statistic: 70.81 on 2 and 117 DF, p-value: < 2.2e-16

**15-30cm depth SOC model**

Call:

lm(formula = nSHS ~ nsilt + nsand + log_flow_acc, data = (farms_points_sf_15_all %>%

group_by(Rep, Farm, Stability) %>% summarise_all(mean) %>%

ungroup %>% dplyr::select(nSHS, ncation, nclay, nsilt, nsand,

Slope, aspect, log_flow_acc)))

Residuals:

Min 1Q Median 3Q Max

-1.76994 -0.45663 0.01706 0.49689 1.98193

Coefficients:

Estimate Std. Error t value Pr(>|t|)

(Intercept) -0.3368 0.1026 -3.284 0.00135 **

nsilt -0.3923 0.1755 -2.235 0.02731 *

nsand -0.2940 0.1679 -1.751 0.08261 .

log_flow_acc 0.4630 0.1053 4.397 2.45e-05 ***

---

Signif. codes:

0 ‘***’ 0.001 ‘**’ 0.01 ‘*’ 0.05 ‘.’ 0.1 ‘ ’ 1

Residual standard error: 0.7469 on 116 degrees of freedom

Multiple R-squared: 0.192, Adjusted R-squared: 0.1711

F-statistic: 9.188 on 3 and 116 DF, p-value: 1.672e-05

### Multilinear regression of SOC stepwise model test of assumptions for 0-30 depth.

Regional

1. independence of Errors and Homoscedasticity:


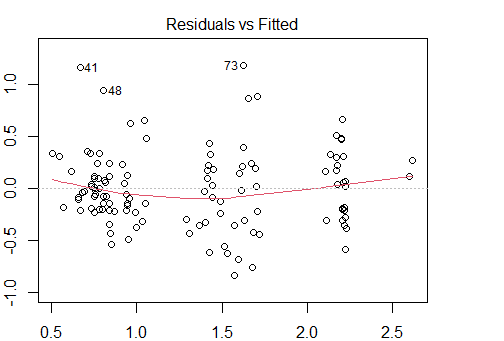


Figure S1 The residual verse fitted plot of the regional SOC model shows no relationship between fitted values and the residuals or the residual variance. The spatial auto correlation of the fitted values is evident.

2. Normality of Residuals:


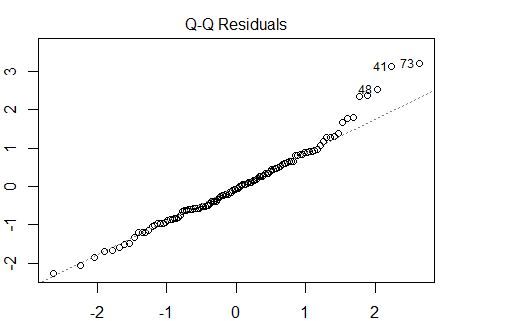


Figure S2 The Q-Q plot of regional SOC residuals demonstrate the residuals are normally distributed.

Local

1. independence of Errors and Homoscedasticity:
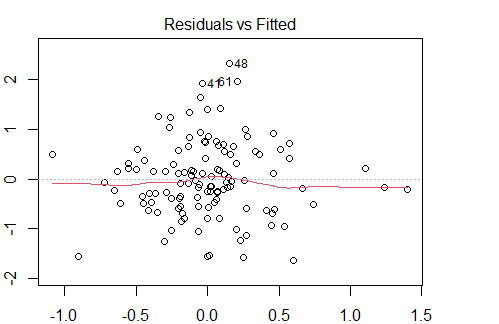


Figure S3. The residual verse fitted plot of the local SOC model shows no relationship between fitted values and the residuals or the residual variance.

2. Normality of Residuals:


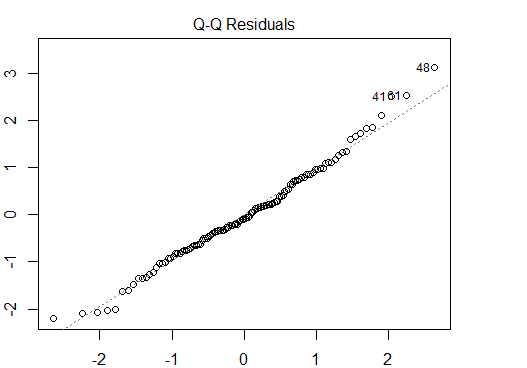


Figure S4. the Q-Q plot of local SOC residuals demonstrates that the residuals are normally distributed.

### Multilinear regression of SHS Regional stepwise model.

**Full depth 0-30cm SHS model**

Start: AIC=282.61

SHS ~ claytotal.r + sandtotal.r + tavg + pr

Df Sum of Sq RSS AIC

- pr 1 1.808 1165.4 280.80

<none> 1163.5 282.61

- sandtotal.r 1 91.469 1255.0 289.69

- tavg 1 117.410 1281.0 292.14

- claytotal.r 1 153.747 1317.3 295.50

Step: AIC=280.79

SHS ~ claytotal.r + sandtotal.r + tavg

Df Sum of Sq RSS AIC

<none> 1165.4 280.80

- sandtotal.r 1 92.843 1258.2 287.99

- tavg 1 118.213 1283.6 290.39

- claytotal.r 1 157.476 1322.8 294.00

Call:

lm(formula = SHS ~ claytotal.r + sandtotal.r + tavg, data = (farms_points_sf_15_all %>%

group_by(Rep, Farm, Stability) %>% summarise_all(mean) %>%

ungroup %>% dplyr::select(SHS, claytotal.r, sandtotal.r,

tavg, pr)))

Residuals:

Min 1Q Median 3Q Max

-10.3193 -1.8897 0.3421 2.3411 8.5649

Coefficients:

Estimate Std. Error t value Pr(>|t|)

(Intercept) -20.87262 10.95683 -1.905 0.059258 .

claytotal.r 0.69458 0.17543 3.959 0.000130 ***

sandtotal.r 0.20766 0.06831 3.040 0.002925 **

tavg 1.91300 0.55768 3.430 0.000836 ***

---

Signif. codes:

0 ‘***’ 0.001 ‘**’ 0.01 ‘*’ 0.05 ‘.’ 0.1 ‘ ’ 1

Residual standard error: 3.17 on 116 degrees of freedom

Multiple R-squared: 0.2967, Adjusted R-squared: 0.2785

F-statistic: 16.32 on 3 and 116 DF, p-value: 6.524e-09

**0-15cm SHS model**

Call:

lm(formula = SHS ~ claytotal.r, data = (farms_points_sf_15_all %>%

filter(Depth_cm == 15) %>% dplyr::select(SHS, claytotal.r,

sandtotal.r, tavg, pr)))

Residuals:

Min 1Q Median 3Q Max

-9.889 -2.164 -0.093 2.039 6.821

Coefficients:

Estimate Std. Error t value Pr(>|t|)

(Intercept) 16.22172 0.70800 22.912 < 2e-16 ***

claytotal.r 0.28270 0.03395 8.326 1.71e-13 ***

---

Signif. codes:

0 ‘***’ 0.001 ‘**’ 0.01 ‘*’ 0.05 ‘.’ 0.1 ‘ ’ 1

Residual standard error: 3.15 on 118 degrees of freedom

Multiple R-squared: 0.3701, Adjusted R-squared: 0.3647

F-statistic: 69.32 on 1 and 118 DF, p-value: 1.712e-13

**15-30cm SHS model**

Call:

lm(formula = SHS ~ claytotal.r + sandtotal.r, data = (farms_points_sf_15_all %>%

filter(Depth_cm == 30) %>% dplyr::select(SHS, claytotal.r,

sandtotal.r, tavg, pr)))

Residuals:

Min 1Q Median 3Q Max

-9.4175 -2.2514 0.1942 2.4011 15.4011

Coefficients:

Estimate Std. Error t value Pr(>|t|)

(Intercept) 27.84626 3.42251 8.136 4.89e-13 ***

claytotal.r -0.20805 0.10082 -2.064 0.04127 *

sandtotal.r -0.10376 0.03208 -3.234 0.00159 **

---

Signif. codes:

0 ‘***’ 0.001 ‘**’ 0.01 ‘*’ 0.05 ‘.’ 0.1 ‘ ’ 1

Residual standard error: 4.007 on 117 degrees of freedom

Multiple R-squared: 0.1155, Adjusted R-squared: 0.1004

F-statistic: 7.641 on 2 and 117 DF, p-value: 0.0007601

### Multilinear regression of SOC local stepwise model.

**Full depth 0-30cm SOC model**

Start: AIC=-8.13

nSOC ~ nclay + nsilt + nsand + Slope + aspect + log_flow_acc

Df Sum of Sq RSS AIC

- nclay 1 0.1347 99.924 -9.9697

- nsilt 1 0.1626 99.952 -9.9362

- nsand 1 0.1730 99.962 -9.9237

- aspect 1 0.3354 100.125 -9.7289

<none> 99.789 -8.1316

- log_flow_acc 1 2.9460 102.735 -6.6402

- Slope 1 6.6532 106.443 -2.3863

Step: AIC=-9.97

nSOC ~ nsilt + nsand + Slope + aspect + log_flow_acc

Df Sum of Sq RSS AIC

- nsand 1 0.0426 99.967 -11.9185

- aspect 1 0.3386 100.263 -11.5638

- nsilt 1 0.7866 100.711 -11.0288

<none> 99.924 -9.9697

- log_flow_acc 1 3.6411 103.565 -7.6748

- Slope 1 7.1846 107.109 -3.6377

Step: AIC=-11.92

nSOC ~ nsilt + Slope + aspect + log_flow_acc

Df Sum of Sq RSS AIC

- aspect 1 0.3378 100.304 -13.5138

<none> 99.967 -11.9185

- nsilt 1 2.2757 102.242 -11.2175

- log_flow_acc 1 3.6288 103.596 -9.6397

- Slope 1 7.4622 107.429 -5.2794

Step: AIC=-13.51

nSOC ~ nsilt + Slope + log_flow_acc

Df Sum of Sq RSS AIC

<none> 100.30 -13.5138

- nsilt 1 2.0485 102.35 -13.0877

- log_flow_acc 1 3.4460 103.75 -11.4604

- Slope 1 7.6790 107.98 -6.6616

Call:

lm(formula = nSOC ~ nsilt + Slope + log_flow_acc, data = (farms_points_sf_15_all %>%

filter(Depth_cm == 30) %>% dplyr::select(nSOC, nclay, nsilt,

nsand, Slope, aspect, log_flow_acc)))

Residuals:

Min 1Q Median 3Q Max

-3.0112 -0.5131 -0.0879 0.5321 3.8882

Coefficients:

Estimate Std. Error t value Pr(>|t|)

(Intercept) -0.21078 0.16922 -1.246 0.21544

nsilt -0.15917 0.10341 -1.539 0.12648

Slope -0.18258 0.06127 -2.980 0.00351 **

log_flow_acc 0.26714 0.13382 1.996 0.04824 *

---

Signif. codes:

0 ‘***’ 0.001 ‘**’ 0.01 ‘*’ 0.05 ‘.’ 0.1 ‘ ’ 1

Residual standard error: 0.9299 on 116 degrees of freedom

Multiple R-squared: 0.1276, Adjusted R-squared: 0.105

F-statistic: 5.656 on 3 and 116 DF, p-value: 0.001187

**0-15cm SOC model**

Call:

lm(formula = nSHS ~ nsilt + nsand + log_flow_acc, data = (farms_points_sf_15_all %>%

filter(Depth_cm == 15) %>% dplyr::select(nSHS, nclay, nsilt,

nsand, Slope, aspect, log_flow_acc)))

Residuals:

Min 1Q Median 3Q Max

-1.97708 -0.58231 0.01321 0.60389 1.70537

Coefficients:

Estimate Std. Error t value Pr(>|t|)

(Intercept) 0.005766 0.109855 0.052 0.9582

nsilt -0.156217 0.078240 -1.997 0.0482 *

nsand -0.195686 0.084321 -2.321 0.0220 *

log_flow_acc 0.465685 0.110220 4.225 4.78e-05 ***

---

Signif. codes:

0 ‘***’ 0.001 ‘**’ 0.01 ‘*’ 0.05 ‘.’ 0.1 ‘ ’ 1

Residual standard error: 0.782 on 116 degrees of freedom

Multiple R-squared: 0.1879, Adjusted R-squared: 0.1669

F-statistic: 8.948 on 3 and 116 DF, p-value: 2.216e-05

**15-30cm SOC model**

Call:

lm(formula = nSHS ~ log_flow_acc, data = (farms_points_sf_15_all %>%

filter(Depth_cm == 30) %>% dplyr::select(nSHS, nclay, nsilt,

nsand, Slope, aspect, log_flow_acc)))

Residuals:

Min 1Q Median 3Q Max

-1.7730 -0.6110 -0.0152 0.5781 3.5286

Coefficients:

Estimate Std. Error t value Pr(>|t|)

(Intercept) -0.6854 0.1288 -5.323 4.93e-07 ***

log_flow_acc 0.4984 0.1317 3.785 0.000243 ***

---

Signif. codes:

0 ‘***’ 0.001 ‘**’ 0.01 ‘*’ 0.05 ‘.’ 0.1 ‘ ’ 1

Residual standard error: 0.9428 on 118 degrees of freedom

Multiple R-squared: 0.1083, Adjusted R-squared: 0.1007

F-statistic: 14.33 on 1 and 118 DF, p-value: 0.0002432

### Multilinear regression of SHS local stepwise model.

**Full depth 0-30cm SHS model**

> lm_local_SHS0_30 =step(lm(nSHS ~., (farms_points_sf_15_all%>% group_by(Rep, Farm,Stability ) %>%

+ summarise_all(mean) %>% ungroup %>%

+ dplyr::select(nSHS, ncation,nclay,nsilt,nsand,Slope,aspect,

+ log_flow_acc))))#, lat, long ))))

Start: AIC=-60.82

nSHS ~ ncation + nclay + nsilt + nsand + Slope + aspect + log_flow_acc

Df Sum of Sq RSS

- aspect 1 0.0017 63.266

- nclay 1 0.0673 63.331

- Slope 1 0.5541 63.818

- ncation 1 0.7756 64.040

- nsand 1 0.8499 64.114

<none> 63.264

- nsilt 1 2.0696 65.334

- log_flow_acc 1 6.7175 69.982

AIC

- aspect -62.818

- nclay -62.693

- Slope -61.774

- ncation -61.359

- nsand -61.220

<none> -60.821

- nsilt -58.958

- log_flow_acc -50.711

Step: AIC=-62.82

nSHS ~ ncation + nclay + nsilt + nsand + Slope + log_flow_acc

Df Sum of Sq RSS

- nclay 1 0.0697 63.336

- Slope 1 0.5699 63.836

- ncation 1 0.7740 64.040

- nsand 1 0.8756 64.141

<none> 63.266

- nsilt 1 2.1278 65.394

- log_flow_acc 1 6.8343 70.100

AIC

- nclay -64.685

- Slope -63.742

- ncation -63.358

- nsand -63.168

<none> -62.818

- nsilt -60.848

- log_flow_acc -52.508

Step: AIC=-64.69

nSHS ~ ncation + nsilt + nsand + Slope + log_flow_acc

Df Sum of Sq RSS

- Slope 1 0.6029 63.938

- ncation 1 0.7611 64.097

<none> 63.336

- nsand 1 1.5203 64.856

- nsilt 1 2.6086 65.944

- log_flow_acc 1 8.0242 71.360

AIC

- Slope -65.549

- ncation -65.252

<none> -64.685

- nsand -63.839

- nsilt -61.842

- log_flow_acc -52.371

Step: AIC=-65.55

nSHS ~ ncation + nsilt + nsand + log_flow_acc

Df Sum of Sq RSS

- ncation 1 0.7755 64.714

<none> 63.938

- nsand 1 1.5098 65.448

- nsilt 1 2.5523 66.491

- log_flow_acc 1 9.5405 73.479

AIC

- ncation -66.102

<none> -65.549

- nsand -64.748

- nsilt -62.851

- log_flow_acc -50.859

Step: AIC=-66.1

nSHS ~ nsilt + nsand + log_flow_acc

Df Sum of Sq RSS

<none> 64.714

- nsand 1 1.7102 66.424

- nsilt 1 2.7876 67.502

- log_flow_acc 1 10.7843 75.498

AIC

<none> -66.102

- nsand -64.972

- nsilt -63.041

- log_flow_acc -49.606

Call:

lm(formula = nSHS ~ nsilt + nsand + log_flow_acc, data = (farms_points_sf_15_all %>%

group_by(Rep, Farm, Stability) %>% summarise_all(mean) %>%

ungroup %>% dplyr::select(nSHS, ncation, nclay, nsilt, nsand,

Slope, aspect, log_flow_acc)))

Residuals:

Min 1Q Median 3Q

-1.76994 -0.45663 0.01706 0.49689

Max

1.98193

Coefficients:

Estimate Std. Error

(Intercept) -0.3368 0.1026

nsilt -0.3923 0.1755

nsand -0.2940 0.1679

log_flow_acc 0.4630 0.1053

t value Pr(>|t|)

(Intercept) -3.284 0.00135 **

nsilt -2.235 0.02731 *

nsand -1.751 0.08261 .

log_flow_acc 4.397 2.45e-05 ***

---

Signif. codes:

0 ‘***’ 0.001 ‘**’ 0.01 ‘*’

0.05 ‘.’ 0.1 ‘ ’ 1

Residual standard error: 0.7469 on 116 degrees of freedom

Multiple R-squared: 0.192, Adjusted R-squared: 0.1711

F-statistic: 9.188 on 3 and 116 DF, p-value: 1.672e-05

**0-15cm SHS model**

Call:

lm(formula = nSHS ~ nsilt + nsand + log_flow_acc, data = (farms_points_sf_15_all %>%

filter(Depth_cm == 15) %>% dplyr::select(nSHS, ncation, nclay,

nsilt, nsand, Slope, aspect, log_flow_acc)))

Residuals:

Min 1Q Median 3Q

-1.97708 -0.58231 0.01321 0.60389

Max

1.70537

Coefficients:

Estimate Std. Error

(Intercept) 0.005766 0.109855

nsilt -0.156217 0.078240

nsand -0.195686 0.084321

log_flow_acc 0.465685 0.110220

t value Pr(>|t|)

(Intercept) 0.052 0.9582

nsilt -1.997 0.0482 *

nsand -2.321 0.0220 *

log_flow_acc 4.225 4.78e-05 ***

---

Signif. codes:

0 ‘***’ 0.001 ‘**’ 0.01 ‘*’

0.05 ‘.’ 0.1 ‘ ’ 1

Residual standard error: 0.782 on 116 degrees of freedom

Multiple R-squared: 0.1879, Adjusted R-squared: 0.1669

F-statistic: 8.948 on 3 and 116 DF, p-value: 2.216e-05

**15-30cm SHS model**

Call:

lm(formula = nSHS ~ log_flow_acc, data = (farms_points_sf_15_all %>%

filter(Depth_cm == 30) %>% dplyr::select(nSHS, ncation, nclay,

nsilt, nsand, Slope, aspect, log_flow_acc)))

Residuals:

Min 1Q Median 3Q

-1.7730 -0.6110 -0.0152 0.5781

Max

3.5286

Coefficients:

Estimate Std. Error

(Intercept) -0.6854 0.1288

log_flow_acc 0.4984 0.1317

t value Pr(>|t|)

(Intercept) -5.323 4.93e-07 ***

log_flow_acc 3.785 0.000243 ***

---

Signif. codes:

0 ‘***’ 0.001 ‘**’ 0.01 ‘*’

0.05 ‘.’ 0.1 ‘ ’ 1

Residual standard error: 0.9428 on 118 degrees of freedom

Multiple R-squared: 0.1083, Adjusted R-squared: 0.1007

F-statistic: 14.33 on 1 and 118 DF, p-value: 0.0002432

### Multilinear regression of SHS stepwise model test of assumptions for the 0-30cm depth.

Regional

1. independence of Errors and Homoscedasticity:


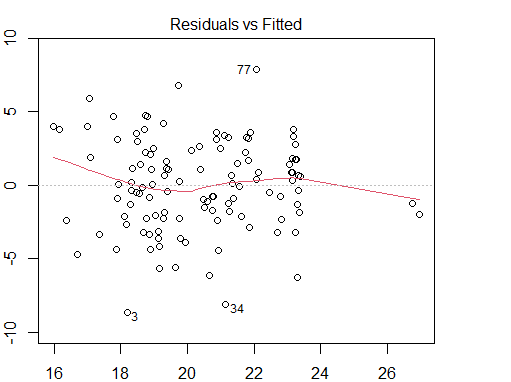


Figure S5 The residual verse fitted plot of the regional SHS model shows no relationship between fitted values and the residuals or the residual variance.

2. Normality of Residuals:


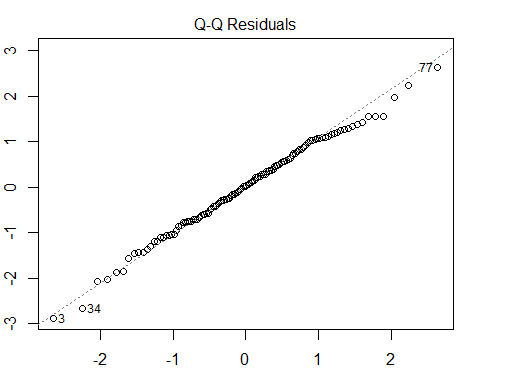


Figure S6 The Q-Q plot of regional SHS residuals demonstrate the residuals are normally distributed.

Local

1. independence of Errors and Homoscedasticity:
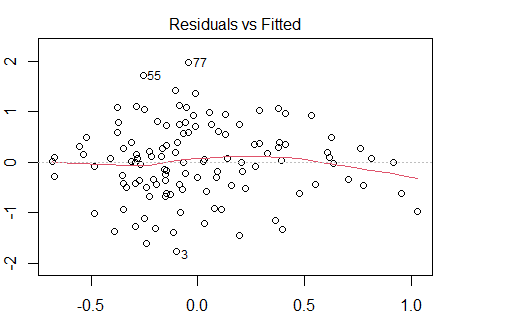


Figure S7. The residual verse fitted plot of the local SOC model shows no relationship between fitted values and the residuals or the residual variance.

2. Normality of Residuals:


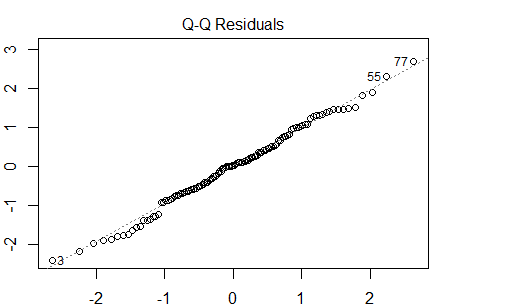


Figure S8. the Q-Q plot of local SHS residuals demonstrate the residuals are normally distributed.

### ANOVA model levenes test.

Field level SOC are statistical equal in variance across YSZ.

Levene's Test for Homogeneity of Variance (center = median)

Df F value Pr(>F)

group 3 1.3845 0.2482

236

Field level SHS are statistical equal in variance across YSZ.

Levene's Test for Homogeneity of Variance (center = median)

Df F value Pr(>F)

group 3 0.388 0.7617

236

# S3. Results figures

Table S1 The absolute average and (standard deviation) of soil organic carbon (SOC) for each yield stability zone (SZ); low and stable (LS), medium and stable (MS), high and stable (HS), and unstable (US) for each field.

|  |  | Zone average, absolute SOC (%) | | | |
| --- | --- | --- | --- | --- | --- |
| State | **Field** | LS | MS | HS | US |
| Michigan | 100_Cort | 0.63(0.14) | 0.69(0.17) | 0.96(0.06) | 1.00(0.31) |
| Michigan | 210_Well | 0.77(0.23) | 0.78(0.33) | 0.87(0.13) | 1.17(0.53) |
| Michigan | ZC1 | 1.09(0.07) | 1.15(0.14) | 1.76(0.33) | 1.73(0.19) |
| Michigan | SR13 | 1.17(0.42) | 1.23(0.09) | 1.21(0.53) | 1.67(0.22) |
| Michigan | 71_ | 0.80(0.26) | 0.75(0.17) | 0.70(0.07) | 1.01(0.30) |
| Michigan | 79_2 | 0.69(0.16) | 0.87(0.11) | 0.84(0.03) | 0.76(0.11) |
| Indiana | Watt East | 1.52(0.24) | 1.86(0.38) | 2.75(0.71) | 2.55(0.34) |
| Indiana | Micic Stateline | 1.33(0.41) | 1.62(0.16) | 1.91(0.42) | 1.11(0.32) |
| Illinois | Horn South | 2.36(0.45) | 2.45(0.08) | 2.78(0.14) | 2.22(0.18) |
| Illinois | South Lane | 2.26(0.12) | 2.31(0.32) | 1.93(0.25) | 2.21(0.15) |


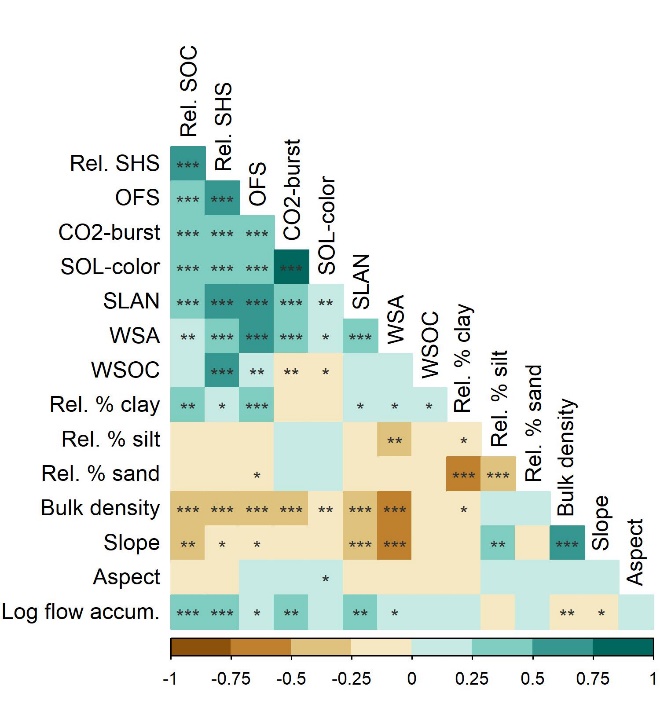

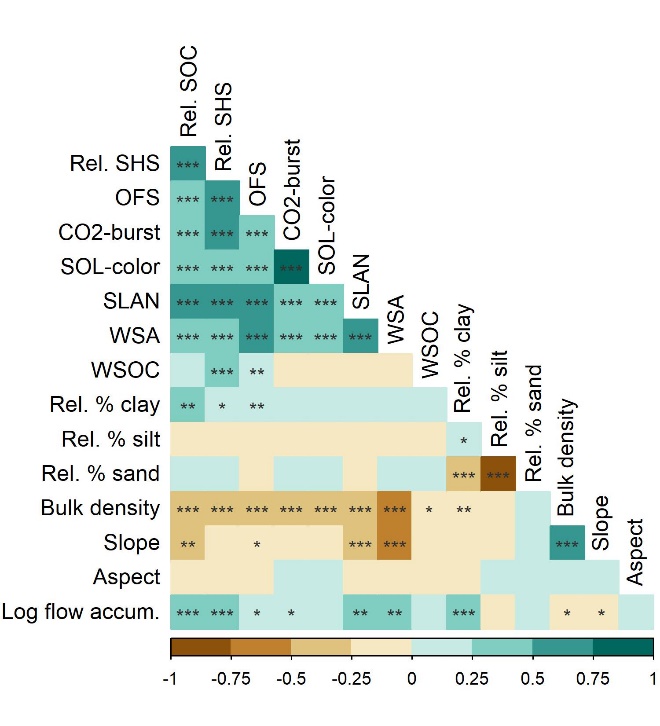


Figure 9. Local scale correlation analysis for depth increments 0-15cm(Left) and 0-30cm (Right.

Figure S9. Local scale correlation analysis for depth increments 0-15 cm (left) and 0-30 cm right


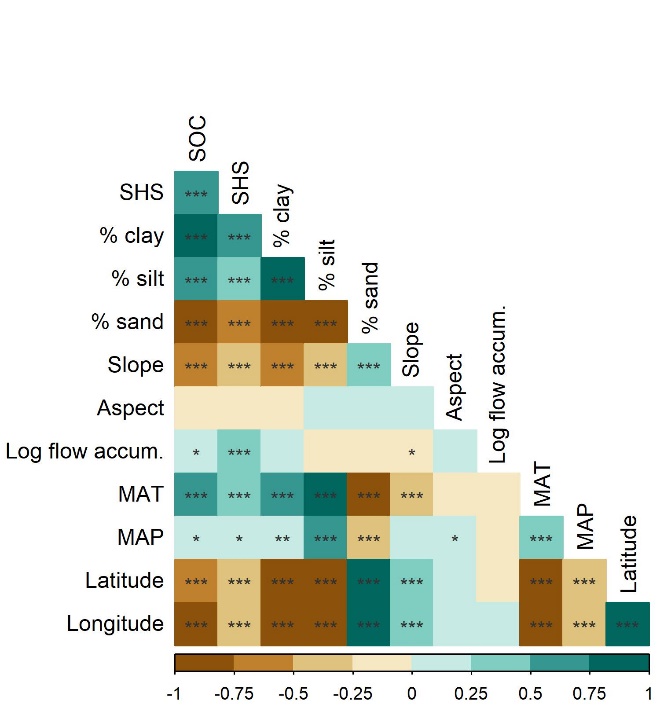

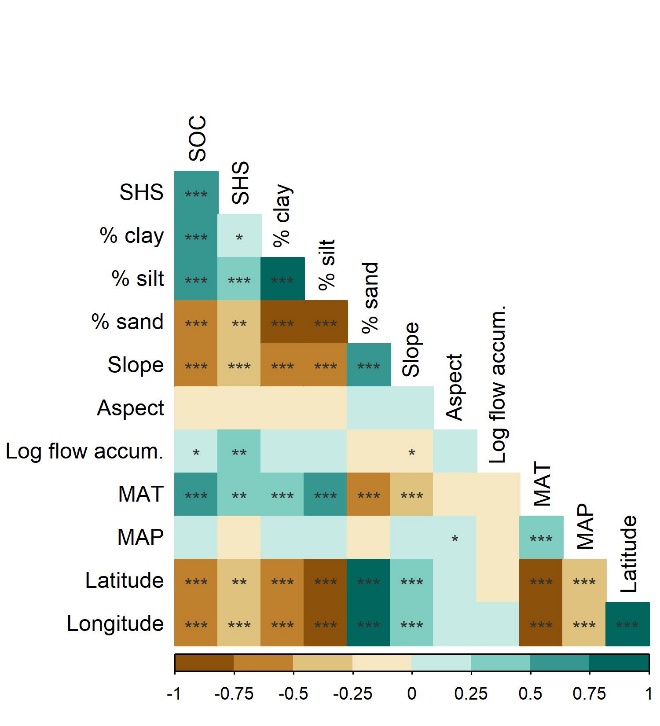


Figure 10 Regional scale correlation analysis for depth increments 0-15cm(Left) and 0-30cm (Right.

Figure S10. Regional scale correlation analysis for depth increments 0-15 cm (left) and 0-30 cm (Right)

## Regional and local correlation matrices

Table S2. Local matrices for 0-30 depth increment samples of correlation values and significance values.


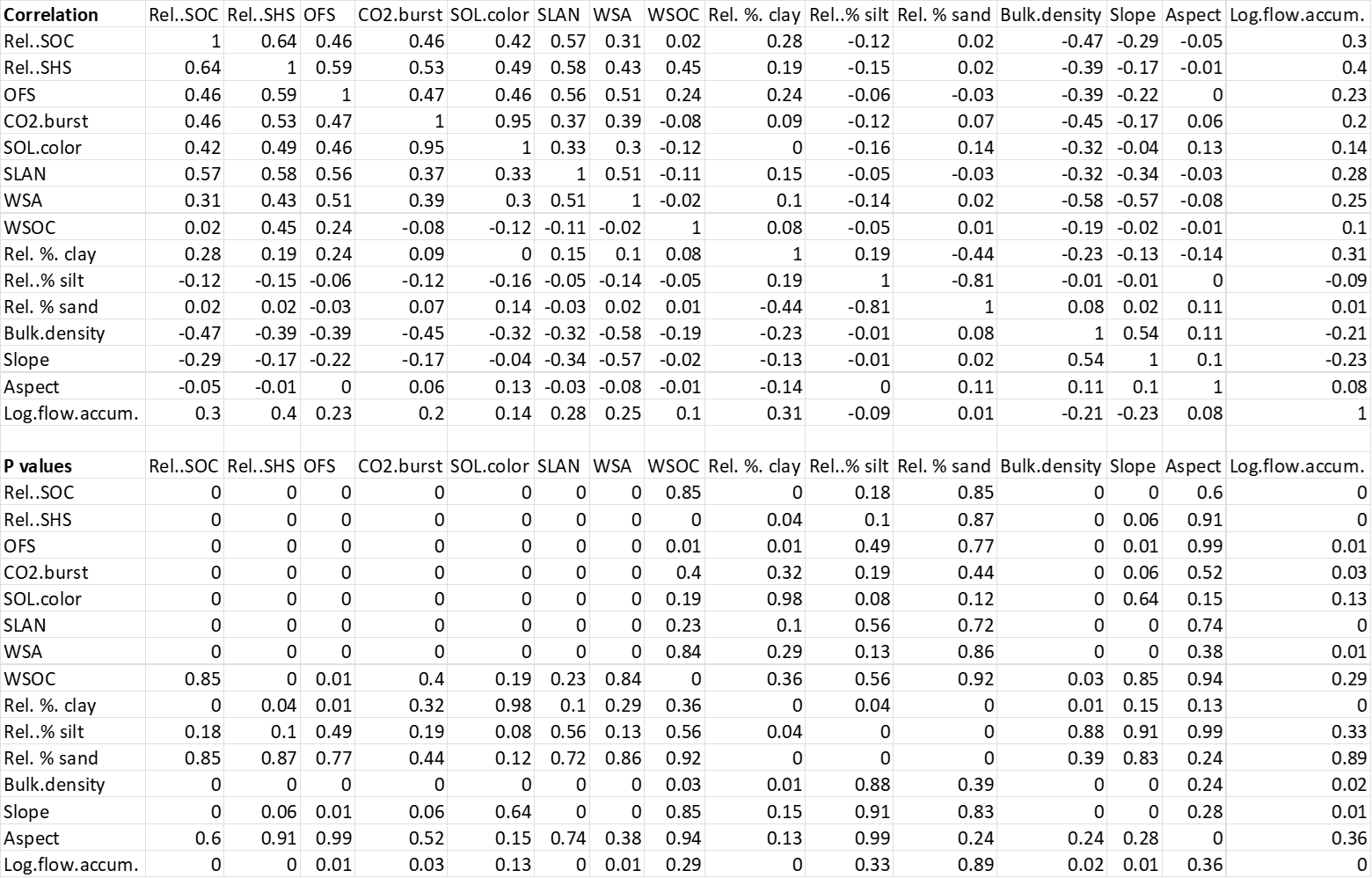


Table S3. Regional matrices for 0-30 depth increment samples of correlation values and significance values.


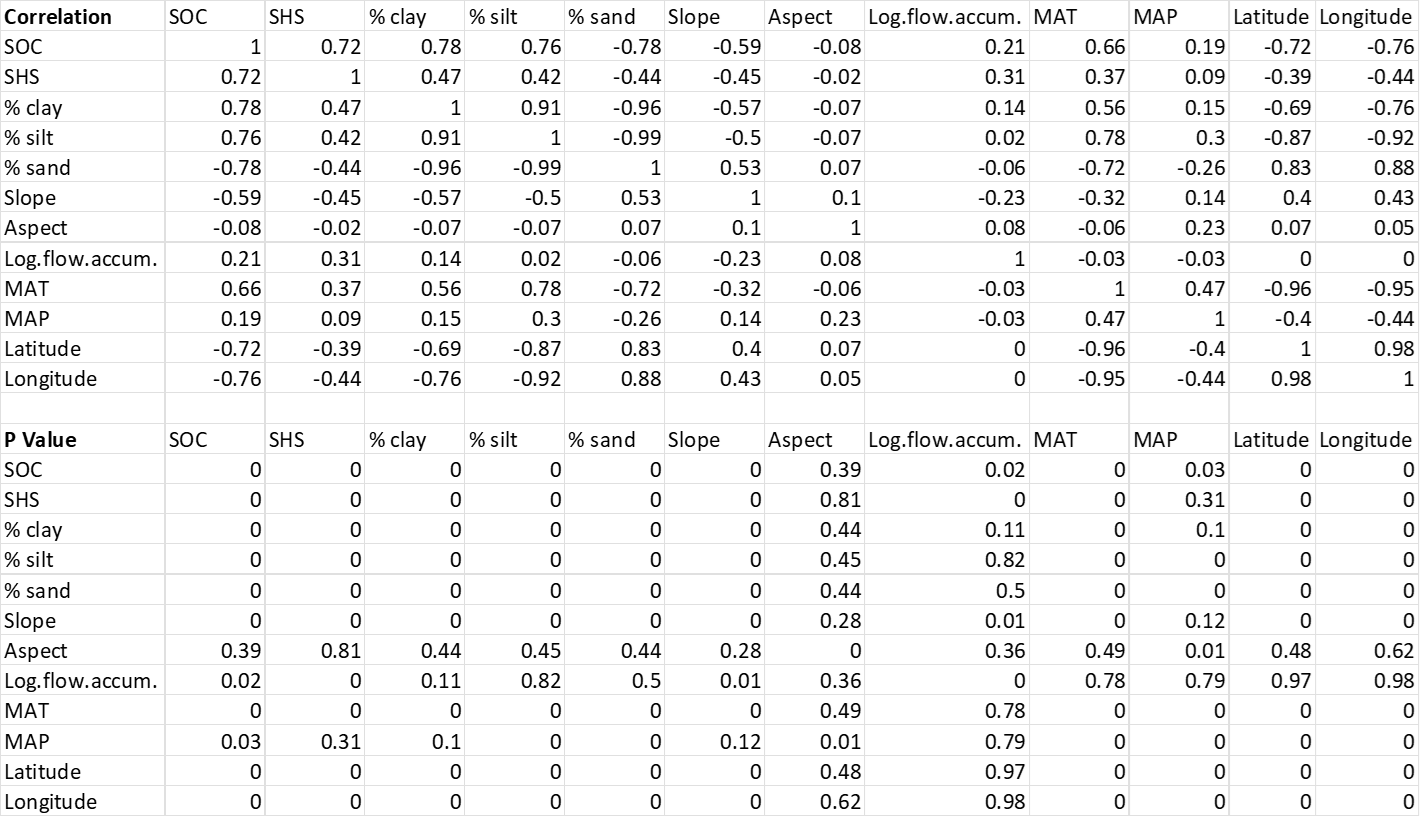


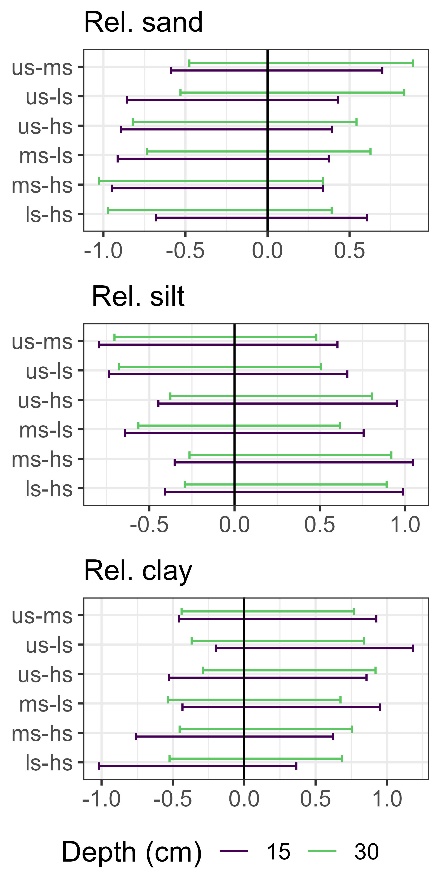

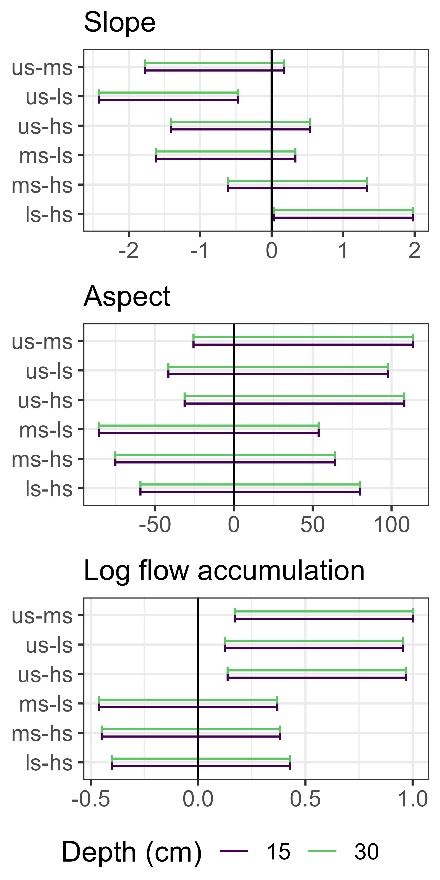

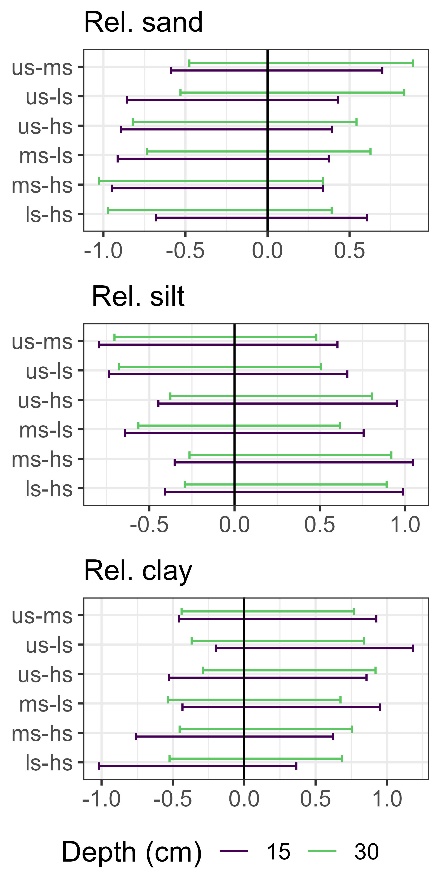

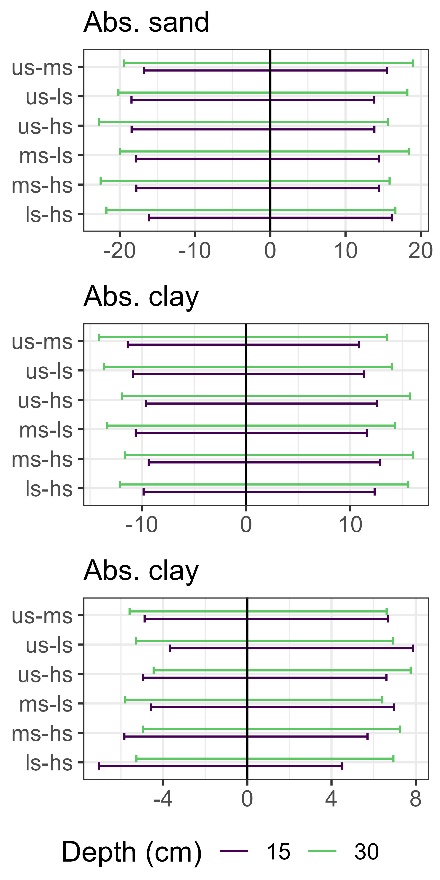


Figure S11. Field level 95% confidence interval ANOVA analysis of yield stability zone (SZ) differences between zones; low and stable (LS), medium and stable (MS), high and stable (HS), and unstable (US) at soil depths of 0-15 cm (15) and 15-30 cm (30) for absolute (Abs.) and relative (Rel.) values of soil texture (clay, silt, sand) and topography (slope, aspect, and log flow accumulation).


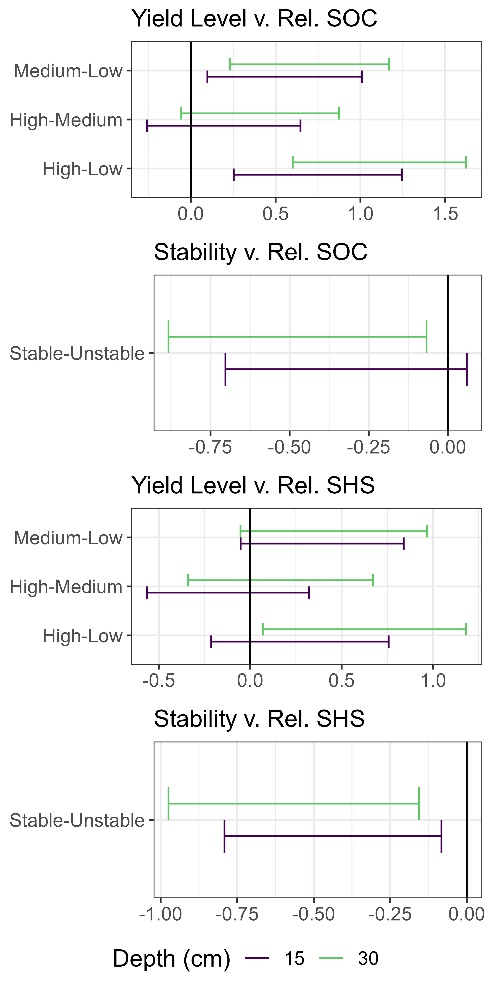


Figure S12 95% confidence interval of difference for yield level and yield stability for soil organic carbon (SOC) and soil health score (SHS).


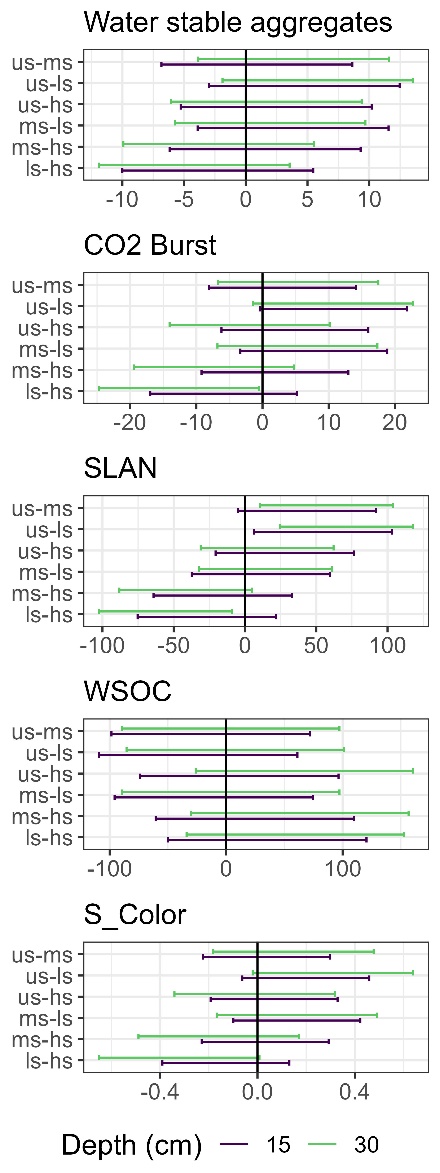


Figure S13 . 95% confidence interval of differences for components for the soil health score (SHS); CO2-burst, Solvita (S) color, Solvita labile amino nitrogen (SLAN), water stable aggregates, and water-soluble organic carbon (WSOC).

# S4. Method: R Code

## Paper Data managment

## authors: Ames Fowler Fidel Muariea

## email: fowler53@msu.edu

##

##

##1 libraries ####

packages = c('tidyverse','lubridate','readxl','ggpubr','usmap','sf','cowplot',

'whitebox','RColorBrewer','corrplot','maps', 'FedData', 'ggspatial')

out = sapply(packages, require,character.only = TRUE)

folders = c('inputs', 'outputs', 'figures')

out = sapply(folders, dir.create)

##2 source data ####

soil_health_set <- read_excel("inputs/MASTER_DATA_MSU-10-Farms_WIll.xlsx",sheet = 'Soil Health Set',skip=2)

log <- read_excel("inputs/MASTER_DATA_MSU-10-Farms_WIll.xlsx",sheet = 'LOG SHEET',skip=1)

BD_df <- read_excel("inputs/MASTER_DATA_MSU-10-Farms_WIll.xlsx",sheet = 'Field Bulk Density',skip=2) %>%

mutate(Depth_cm = Depth %>% strsplit(.,"-") %>% sapply(.,"[",3))

names(soil_health_set) =make.names(names(soil_health_set))

soil_health_set[3:6] = lapply(soil_health_set[3:6], as.factor)

soil_health_set$Stability_number = ifelse(soil_health_set$Stability == 'us',1,

ifelse(soil_health_set$Stability=='ls',2,

ifelse(soil_health_set$Stability=='ms', 3,

4)))

soil_health_set$Depth_cm = ifelse(as.character(soil_health_set$Depth) == 'top-0-6',15,30)

log_simple = log %>% dplyr::select(Farm,Stability= Code, Lat, Long, Rep, New_code) %>%

unique() %>% mutate(Rep = ifelse(Rep== 1,"A", ifelse(Rep==2,"B", "C")))

soil_health_set = soil_health_set %>% left_join(log_simple) %>%

mutate(level_code = New_code %>% strsplit("") %>% sapply(.,"[", 1) %>%

factor( levels = c("l","m","h"),labels = c("Low", "Medium", "High")),

stability_code = New_code %>% strsplit("") %>% sapply(.,"[", 2)%>%

factor( levels = c("u", "s"), labels = c("Unstable", "Stable" )))

##3 site map - F1 ####

if(!dir.exists("./figures/paper_figs_231011/")){

dir.create("./figures/paper_figs_231011/")

}

#### sitemap library(usmap)

#get state maps

conus <- maps::map("usa", plot = FALSE, fill = TRUE) %>% st_as_sf %>% filter(ID == "main")

states <- st_as_sf(maps::map("state", plot = FALSE, fill = TRUE)) %>% filter(ID %in% c("michigan", "wisconsin", "illinois","indiana"))

# Plotting

# sites <- ggplot( )+# %>% filter(ID %in% c("michigan", "wisconsin", "illinois","indiana"))) +

conus_plot <- geom_sf(data = conus, fill = NA, alpha = .25)

states_plot <- geom_sf(data = states, fill = "lightgreen", alpha = .25)

sites_plot <- geom_jitter(data = temp, aes(x = Long, y = Lat, fill = Farm %>%

factor(levels = c("SR13","ZC1", "1-Cort","2-Well", "F-79-2",

"F-71","Watt-East","Micic-Stateline",

"Horn-South","South-Lane"))),

size = 4, shape = 23, width = .20,

height = .20, alpha = .5)

a <- ggplot()+

conus_plot+states_plot +

theme_void()

b <- ggplot()+

states_plot+sites_plot +theme_void()+labs(fill = "Field") +

ggspatial::annotation_scale(location = "br") +

annotation_north_arrow(which_north = "grid", location = "bl")

seed = 1000

g <- ggdraw(b) +

draw_plot(

{ a +theme(legend.position = "none")

},

# The distance along a (0,1) x-axis to draw the left edge of the plot

x = .55,

# The distance along a (0,1) y-axis to draw the bottom edge of the plot

y = .70,

# The width and height of the plot expressed as proportion of the entire ggdraw object

width = 0.4,

height = 0.4)

ggsave(g, filename = "./figures/paper_figs_231011/sitemap.jpeg", height = 5, width = 5, dpi = 700)

##4 site climate data ####

temp = soil_health_set %>%

group_by(Farm) %>%

summarize (Lat = first(Lat), Long = first(Long))

farms_sf <- st_as_sf(temp, coords = c("Long", "Lat"), crs = st_crs(4326))

farms_points_sf<- st_as_sf(soil_health_set, coords = c("Long", "Lat"),

crs = st_crs(4326)) %>% split(.$Farm)

if(!file.exists("./outputs/temp_pr_20yrnormal")){

clim_list = as.list(rep(NA, nrow(farms_sf)))

test <- for(k in seq(1:nrow(farms_sf))){

H=farms_sf[k,]

temp <- climateR::getGridMET(AOI = (H), varname = c("pr","tmmn","tmmx"),

startDate = as.Date("2000-01-01"),

endDate = as.Date("2020-12-31"))

test1 = temp %>% group_by(year(date)) %>%

summarise(pr = sum(pr), tavg = mean((tmmn+tmmx)/2)-273.15) %>%

ungroup() %>%

summarise(pr = mean(pr), tavg = mean(tavg))

gc()

closeAllConnections()

clim_list[[k]] = as.data.frame(cbind(test1, as.data.frame(H)))

}

test_all = clim_list %>% do.call(rbind,.)

saveRDS(test_all,file = "./outputs/temp_pr_20yrnormal")

}else{

test_all = readRDS(file = "./outputs/temp_pr_20yrnormal")

}

MAP = range(test_all$pr) %>% round(0)

MAT = range(test_all$tavg) %>% round(1)

##5 site soil data #####

if(!file.exists("./outputs/soil_texture_out")){

tempSoilOut = purrr::map(farms_points_sf, function(G){

G_15 = G %>% filter(Depth_cm==15)

G_30 = G %>% filter(Depth_cm==30)

tempsoil <- FedData::get_ssurgo(template =as_Spatial(G_15%>% st_buffer(10)), force.redo = T,

label = G$Farm %>% unique(),raw.dir = "./RAW/SSURGO/",

extraction.dir = file.path("EXTRACTIONS",G$Farm %>% unique(), "SSURGO"))

data_temp_15 = terra::intersect(vect(G_15), vect(tempsoil$spatial))

data_temp_30 = terra::intersect(vect(G_30), vect(tempsoil$spatial))

temp = tempsoil$tabular$component %>% filter(mukey %in% unique(data_temp_15$MUKEY), majcompflag=="Yes") %>%

dplyr::select(comppct.r, compname, taxorder, majcompflag, cokey, mukey)

temp_0_15 = tempsoil$tabular$chorizon %>% filter(cokey %in% unique(temp$cokey), hzdept.r<15) %>%

group_by(cokey) %>% mutate(hzdepb.r = ifelse(hzdepb.r>15, 15, hzdepb.r),

hzthk.r = ifelse(hzdepb.r>15, hzdept.r-15, hzthk.r)

) %>% summarize(silttotal.r = sum(silttotal.r *hzthk.r)/sum(hzthk.r),

sandtotal.r = sum(sandtotal.r*hzthk.r)/sum(hzthk.r),

claytotal.r = sum(claytotal.r*hzthk.r)/sum(hzthk.r)) %>%

ungroup() %>% mutate(sumcheck = silttotal.r + sandtotal.r + claytotal.r)

data_ssurgo_15 = temp %>% full_join(temp_0_15) %>% group_by(mukey) %>%

summarise(silttotal.r = sum(silttotal.r*comppct.r)/sum(comppct.r),

sandtotal.r = sum(sandtotal.r*comppct.r)/sum(comppct.r),

claytotal.r = sum(claytotal.r*comppct.r)/sum(comppct.r))

data_temp_15 = data_temp_15 %>%

st_as_sf() %>% rename(mukey = MUKEY) %>% mutate(mukey = mukey %>% as.numeric()) %>%

left_join(data_ssurgo_15) %>% vect

temp_15_30 = tempsoil$tabular$chorizon %>% filter(cokey %in% unique(temp$cokey), hzdept.r>=15) %>%

group_by(cokey) %>% mutate(hzdepb.r = ifelse(hzdept.r<15, 15, hzdept.r),

hzthk.r = ifelse(hzdept.r<15, 15-hzdepb.r, hzthk.r),

hzdepb.r = ifelse(hzdepb.r>30, 30, hzdepb.r),

hzthk.r = ifelse(hzdepb.r>30, hzdept.r-30, hzthk.r)

) %>% summarize(silttotal.r = sum(silttotal.r *hzthk.r)/sum(hzthk.r),

sandtotal.r = sum(sandtotal.r*hzthk.r)/sum(hzthk.r),

claytotal.r = sum(claytotal.r*hzthk.r)/sum(hzthk.r)) %>%

ungroup() %>% mutate(sumcheck = silttotal.r + sandtotal.r + claytotal.r)

data_ssurgo_30 = temp %>% full_join(temp_15_30) %>% group_by(mukey) %>%

summarise(silttotal.r = sum(silttotal.r*comppct.r)/sum(comppct.r),

sandtotal.r = sum(sandtotal.r*comppct.r)/sum(comppct.r),

claytotal.r = sum(claytotal.r*comppct.r)/sum(comppct.r))

data_temp_30 = data_temp_30 %>%

st_as_sf() %>% rename(mukey = MUKEY) %>% mutate(mukey = mukey %>% as.numeric()) %>%

left_join(data_ssurgo_30) %>% vect

return(rbind(data_temp_15, data_temp_30))

})

test_all_soil = tempSoilOut %>% lapply(., st_as_sf) %>% do.call(rbind,.)

saveRDS(test_all_soil,file = "./outputs/soil_texture_out")

}else{

test_all_soil = readRDS(file = "./outputs/soil_texture_out")

}

##6 site table 1 ####

clim_temp = test_all%>% dplyr::select(c("Field" =Farm, 'MAP (MM)' = pr ,

"MAT (C)" = tavg )) %>%

mutate(`MAP (MM)` =`MAP (MM)` %>% round,

`MAT (C)` =`MAT (C)` %>% round(1))

soil_temp = test_all_soil %>% st_drop_geometry() %>%

dplyr::select("Field" =Farm,

'Clay (%)' = claytotal.r ,

"Silt (%)" = silttotal.r,

"Sand (%)" = sandtotal.r ) %>%

group_by(Field) %>% summarise_all(mean)%>%

mutate(`Clay (%)` =`Clay (%)` %>% round(1),

`Silt (%)` =`Silt (%)` %>% round(1),

`Sand (%)` =`Sand (%)` %>% round(1))

Table1_out = merge(clim_temp, soil_temp) %>% arrange(`MAT (C)`)

write.csv(Table1_out, file = "./outputs/Table1_clim_soil.csv")

clayRange = range(Table1_out$`Clay (%)`)

## 7 white box function ####

whitebox_flow_funciton = function(demfile){

whitebox::wbt_init()

dem <- demfile #("./processed_data/KBS_DEM.tif")

# wbt_feature_preserving_smoothing(

# dem = dem,

# output = "./processed_data/KBS_DEM_smoothed.tif",

# filter = 9

# )

DEM_breached_file = file.path(dirname(demfile), "DEM_breached.tif")

Flow_accum_file = file.path(dirname(demfile), "Flow_accum.tif")

Slope_file = file.path(dirname(demfile), "Slope.tif")

TWI_file = file.path(dirname(demfile), "TWI.tif")

DEM_breached = wbt_breach_depressions(dem = demfile,

output = DEM_breached_file)

Flow_accum = wbt_d_inf_flow_accumulation(input = DEM_breached_file,

output = Flow_accum_file, out_type = 'cells')

Slope = wbt_slope(dem = DEM_breached_file,

output = Slope_file,

units = "degrees")

TWI = wbt_wetness_index(sca = Flow_accum_file,

slope = Slope_file,

output = TWI_file)

outraster = c(rast(DEM_breached_file),

rast(Flow_accum_file),

rast(Slope_file),

rast(TWI_file))

}

## 8 Site DEM data ####

if(!file.exists("./outputs/farms_points_sf_15_all")){

DEMOut = purrr::map(tempSoilOut, function(G){

dem_temp <- FedData::get_ned(template =as_Spatial(G %>% st_as_sf %>% st_buffer(50)),res = '13',

force.redo = T,extraction.dir = file.path("EXTRACTIONS",G$Farm %>% unique(), "NED"),

label = G$Farm %>% unique())

demfile = dem_temp@ptr$filenames()

tempslope = terra::terrain(dem_temp, v=c("aspect"))

flowrast = whitebox_flow_funciton(demfile = demfile)

names(dem_temp) ="DEM"

net_dem_temp = dem_temp-min(values(dem_temp))

names(net_dem_temp) = "Net_DEM"

out_raster <- c( dem_temp,net_dem_temp, tempslope, flowrast)

out_raster_points = terra::extract(out_raster, (G))

return(cbind(G,out_raster_points))})

farms_points_sf_15_all = DEMOut %>% lapply(.,as.data.frame) %>% data.table::rbindlist()

saveRDS(farms_points_sf_15_all,file = "./outputs/farms_points_sf_15_all")

}else{

farms_points_sf_15_all = readRDS(file = "./outputs/farms_points_sf_15_all")

}

farms_points_sf_15_all = farms_points_sf_15_all %>% group_by(Farm) %>%

mutate(Net_DEM_percent = Net_DEM/max(Net_DEM),

Flow_accum_log_percent = log(Flow_accum)/max(log(Flow_accum)),

Slope_zscore = (Slope-mean(Slope))/sd(Slope),

# SOC_mean = mean(soc),

# SOC_sd = sd(soc),

nSOC = scale(soc),

#-SOC_mean)/SOC_sd,

# SHS_mean = mean(SHS),

# SHS_sd = sd(SHS),

# ncation = scale(Cation),

nSHS = scale(SHS),

nclay = scale(claytotal.r),

nsand = scale(sandtotal.r),

nsilt = scale(silttotal.r)) %>% ungroup()

# nSHS = (SHS-SHS_mean)/SHS_sd)

farms_points_sf_15_all = left_join(farms_points_sf_15_all%>% mutate(log_flow_acc = log10(Flow_accum)) ,

test_all %>%

mutate(lat =(geometry %>% sf::st_coordinates())[,2],

long = (geometry %>% sf::st_coordinates())[,1]) %>%

dplyr::select(pr, tavg, Farm, lat, long))

farms_sf_mean = farms_points_sf_15_all %>% group_by(Farm) %>% summarise_all(~mean(.x, na.rm=T)) %>% ungroup()

## 9 Regional antibiotic step wise analysis and Corrpolt F2 #####

lm_enviro_SOC0_30 =step(lm(soc ~., (farms_points_sf_15_all%>% group_by(Rep, Farm,Stability ) %>%

summarise_all(mean) %>% ungroup %>%

dplyr::select(soc, claytotal.r,sandtotal.r,#Slope,aspect,

#log_flow_acc,

tavg, pr))))#, lat, long ))))

summary(lm_enviro_SOC0_30)

lm_enviro_SOC0_15 =step(lm(soc ~., (farms_points_sf_15_all%>% filter(Depth_cm==15) %>%

dplyr::select(soc, claytotal.r,sandtotal.r,#Slope,aspect,

#log_flow_acc,

tavg, pr))))#, lat, long ))))

summary(lm_enviro_SOC0_15)

lm_enviro_SOC15_30 =step(lm(soc ~., (farms_points_sf_15_all%>% filter(Depth_cm==30) %>%

dplyr::select(soc, claytotal.r,sandtotal.r,#Slope,aspect,

#log_flow_acc,

tavg, pr))))#, lat, long ))))

summary(lm_enviro_SOC15_30)

lm_enviro_SHS0_30 =step(lm(SHS ~., (farms_points_sf_15_all %>% group_by(Rep, Farm,Stability ) %>%

summarise_all(mean) %>% ungroup %>%

dplyr::select(SHS, claytotal.r,sandtotal.r,#Slope,aspect,

#log_flow_acc,

tavg, pr))))#, lat, long ))))

summary(lm_enviro_SHS0_30)

enviro_vars = farms_points_sf_15_all %>% group_by(Rep, Farm,Stability ) %>%

summarise_all(mean) %>% ungroup %>%

dplyr::select(soc, SHS, claytotal.r,silttotal.r,sandtotal.r,Slope,aspect,

log_flow_acc, tavg, pr, lat, long )

names(enviro_vars) = c("SOC","SHS", "% clay", "% silt" ,"% sand",

"Slope", "Aspect", "Log flow accum.", "MAT",

"MAP", "Latitude", "Longitude")

testRes = cor.mtest(enviro_vars, conf.level = 0.95)

M =cor(enviro_vars)

write_csv(data.frame(M %>% round(2)),

"./figures/paper_figs_231011/regional_corr_mat.csv")

write_csv(data.frame(testRes$p %>% round(2)),

"./figures/paper_figs_231011/regional_corr_mat_P.csv")

jpeg(filename = "./figures/paper_figs_231011/regional_abiotic_corr.jpeg",

width = 5,height = 5.5, units = "in", res=300)

corrplot(M, p.mat = testRes$p, method = 'color', diag = FALSE, type = 'lower',

sig.level = c(0.001, 0.01, 0.05), pch.cex = 0.9, col=brewer.pal(n=8, name="BrBG"),

insig = 'label_sig', pch.col = 'grey20',order = 'original',tl.col = 'black')

dev.off()

#15

enviro_vars_0015 = farms_points_sf_15_all %>% filter(Depth_cm==15) %>%

dplyr::select(soc, SHS, claytotal.r,silttotal.r,sandtotal.r,Slope,aspect,

log_flow_acc, tavg, pr, lat, long )

names(enviro_vars_0015) = c("SOC","SHS", "% clay", "% silt" ,"% sand",

"Slope", "Aspect", "Log flow accum.", "MAT",

"MAP", "Latitude", "Longitude")

testRes = cor.mtest(enviro_vars_0015, conf.level = 0.95)

M =cor(enviro_vars_0015)

write_csv(data.frame(M %>% round(2)),

"./figures/paper_figs_231011/regional_corr_0015_mat.csv")

write_csv(data.frame(testRes$p %>% round(2)),

"./figures/paper_figs_231011/regional_corr_0015_mat_P.csv")

jpeg(filename = "./figures/paper_figs_231011/regional_abiotic_corr0015.jpeg",

width = 5,height = 5.5, units = "in", res=300)

corrplot(M, p.mat = testRes$p, method = 'color', diag = FALSE, type = 'lower',

sig.level = c(0.001, 0.01, 0.05), pch.cex = 0.9, col=brewer.pal(n=8, name="BrBG"),

insig = 'label_sig', pch.col = 'grey20',order = 'original',tl.col = 'black')

dev.off()

#30

enviro_vars_1530 = farms_points_sf_15_all %>% filter(Depth_cm==30) %>%

dplyr::select(soc, SHS, claytotal.r,silttotal.r,sandtotal.r,Slope,aspect,

log_flow_acc, tavg, pr, lat, long )

names(enviro_vars_1530) = c("SOC","SHS", "% clay", "% silt" ,"% sand",

"Slope", "Aspect", "Log flow accum.", "MAT",

"MAP", "Latitude", "Longitude")

testRes = cor.mtest(enviro_vars_1530, conf.level = 0.95)

M =cor(enviro_vars_1530)

write_csv(data.frame(M %>% round(2)),

"./figures/paper_figs_231011/regional_corr_1530_mat.csv")

write_csv(data.frame(testRes$p %>% round(2)),

"./figures/paper_figs_231011/regional_corr_1530_mat_P.csv")

jpeg(filename = "./figures/paper_figs_231011/regional_abiotic_corr1530.jpeg",

width = 5,height = 5.5, units = "in", res=300)

corrplot(M, p.mat = testRes$p, method = 'color', diag = FALSE, type = 'lower',

sig.level = c(0.001, 0.01, 0.05), pch.cex = 0.9, col=brewer.pal(n=8, name="BrBG"),

insig = 'label_sig', pch.col = 'grey20',order = 'original',tl.col = 'black')

dev.off()

# 10 Local linear stepwise models ####

lm_local_SOC0_30 =step(lm(nSOC ~., (farms_points_sf_15_all %>% group_by(Rep, Farm,Stability ) %>%

summarise_all(mean) %>% ungroup %>%

dplyr::select(nSOC, nclay,nsilt,nsand,Slope,aspect,

log_flow_acc))))#, lat, long ))))

summary(lm_local_SOC0_30)

lm_local_SOC0_15 =step(lm(nSOC ~., (farms_points_sf_15_all%>% filter(Depth_cm ==15) %>%

dplyr::select(nSOC, nclay,nsilt,nsand,Slope,aspect,

log_flow_acc))))#, lat, long ))))

summary(lm_local_SOC0_15)

lm_local_SOC15_30 =step(lm(nSOC ~., (farms_points_sf_15_all%>% filter(Depth_cm ==30) %>%

dplyr::select(nSOC, nclay,nsilt,nsand,Slope,aspect,

log_flow_acc))))#, lat, long ))))

summary(lm_local_SOC15_30)

lm_local_SHS0_30 =step(lm(nSHS ~., (farms_points_sf_15_all%>% group_by(Rep, Farm,Stability ) %>%

summarise_all(mean) %>% ungroup %>%

dplyr::select(nSHS, nclay,nsilt,nsand,Slope,aspect,

log_flow_acc))))#, lat, long ))))

summary(lm_local_SHS0_30)

lm_local_SHS0_15 =step(lm(nSHS ~., (farms_points_sf_15_all%>% filter(Depth_cm ==15) %>%

dplyr::select(nSHS, nclay,nsilt,nsand,Slope,aspect,

log_flow_acc))))#, lat, long ))))

summary(lm_local_SHS0_15)

lm_local_SHS15_30 =step(lm(nSHS ~., (farms_points_sf_15_all%>% filter(Depth_cm ==30) %>%

dplyr::select(nSHS, nclay,nsilt,nsand,Slope,aspect,

log_flow_acc))))#, lat, long ))))

summary(lm_local_SHS15_30)

## 11 local Corr plots ####

local_vars = farms_points_sf_15_all%>% group_by(Rep, Farm,Stability ) %>%

summarise_all(mean) %>% ungroup %>% #filter(Depth_cm==15) %>%

dplyr::select(nSOC,nSHS,OFS, CO2, S_Color, SLAN, wsa, WSOC, nclay, nsilt,nsand,BD,Slope,aspect,

log_flow_acc)

names(local_vars) = c("Rel. SOC","Rel. SHS","OFS","CO2-burst", "SOL-color",

"SLAN", "WSA", "WSOC", "Rel. % clay","Rel. % silt",

"Rel. % sand","Bulk density","Slope", "Aspect", "Log flow accum.")

local_vars_15 = farms_points_sf_15_all %>% ungroup() %>% filter(Depth_cm==15) %>%

dplyr::select(nSOC,nSHS,OFS, CO2, S_Color, SLAN, wsa, WSOC, nclay, nsilt,nsand,BD,Slope,aspect,

log_flow_acc)

names(local_vars_15) = c("Rel. SOC","Rel. SHS","OFS","CO2-burst", "SOL-color",

"SLAN", "WSA", "WSOC", "Rel. % clay","Rel. % silt",

"Rel. % sand","Bulk density","Slope", "Aspect", "Log flow accum.")

local_vars_30 = farms_points_sf_15_all %>% ungroup() %>% filter(Depth_cm==30) %>%

dplyr::select(nSOC,nSHS,OFS, CO2, S_Color, SLAN, wsa, WSOC, nclay, nsilt,nsand,BD,Slope,aspect,

log_flow_acc)

names(local_vars_30) = c("Rel. SOC","Rel. SHS","OFS","CO2-burst", "SOL-color",

"SLAN", "WSA", "WSOC", "Rel. % clay","Rel. % silt",

"Rel. % sand","Bulk density","Slope", "Aspect", "Log flow accum.")

testRes = cor.mtest(local_vars, conf.level = 0.95)

M =cor(local_vars)

write_csv(data.frame(M %>% round(2)),

"./figures/paper_figs_231011/local_corr_mat.csv")

write_csv(data.frame(testRes$p %>% round(2)),

"./figures/paper_figs_231011/local_corr_mat_P.csv")

jpeg(filename = "./figures/paper_figs_231011/local_abiotic_corr_0-30CM.jpeg",

width = 5,height = 5.5, units = "in", res=300)

corrplot(M, p.mat = testRes$p, method = 'color', diag = FALSE, type = 'lower',order = 'original',

sig.level = c(0.001, 0.01, 0.05), pch.cex = 0.9, col=brewer.pal(n=8, name="BrBG"),

insig = 'label_sig', pch.col = 'grey20', tl.col = 'black')

dev.off()

testRes = cor.mtest(local_vars_15, conf.level = 0.95)

M =cor(local_vars_15)

write_csv(data.frame(M %>% round(2)),

"./figures/paper_figs_231011/local_corr_0015_mat.csv")

write_csv(data.frame(testRes$p %>% round(2)),

"./figures/paper_figs_231011/local_corr_0015_mat_P.csv")

jpeg(filename = "./figures/paper_figs_231011//local_abiotic_corr_0-15cm.jpeg",

width = 5,height = 5.5, units = "in", res=300)

corrplot(M, p.mat = testRes$p, method = 'color', diag = FALSE, type = 'lower',

sig.level = c(0.001, 0.01, 0.05), pch.cex = 0.9, col=brewer.pal(n=8, name="BrBG"),

insig = 'label_sig', pch.col = 'grey20',order = 'original',tl.col = 'black')

dev.off()

testRes = cor.mtest(local_vars_30, conf.level = 0.95)

M =cor(local_vars_30)

write_csv(data.frame(M %>% round(2)),

"./figures/paper_figs_231011/local_corr_0030_mat.csv")

write_csv(data.frame(testRes$p %>% round(2)),

"./figures/paper_figs_231011/local_corr_0030_mat_P.csv")

jpeg(filename = "./figures/paper_figs_231011//local_abiotic_corr.jpeg",

width = 5,height = 5.5, units = "in", res=300)

corrplot(M, p.mat = testRes$p, method = 'color', diag = FALSE, type = 'lower',

sig.level = c(0.001, 0.01, 0.05), pch.cex = 0.9, col=brewer.pal(n=8, name="BrBG"),

insig = 'label_sig', pch.col = 'grey20', order = 'original',tl.col = 'black')

dev.off()

##12 tuky functions

TUKEY_2_dt = function(TUKEY){

B<-as.data.frame(TUKEY[1])

colnames(B)[2:4]<-c("min",

"max",

"p")

C<-data.frame(id=row.names(B),

min=B$min,

max=B$max,

idt=ifelse(B$p<0.05,

"significant",

"not significant"))

}

TUKEY_2_plot = function(TUKEY, main){

D<- TUKEY %>%

ggplot(aes(id, col = depth %>% factor))+ #linewidth = idt,

geom_errorbar(aes(ymin=min,

ymax=max),

width = 0.5,

# linewidth=1.25,

position=position_dodge(width=0.5))+

geom_hline(yintercept = 0)+

labs(x=NULL, col = "Depth (cm)", title = main)+

coord_flip()+theme_bw()+

theme(text = element_text(size = 14))+

theme(plot.title = element_text(size = 14))+

scale_color_manual(values= viridis::viridis(5)[c(1,4)])

}

dpeth_tukey_plot = function(data = soil_health_delta,x = "Stability", y = "nSOC", title ){

formula_1 = as.formula(sprintf("%s ~ %s", y, x))

m_6 = aov(lm(formula_1, data=data %>% filter(Depth_cm==15)))#group_by(UID, Stability) %>% summarize(nSOC = mean(nSOC))))

TUKEY_6 <- TukeyHSD(x=m_6)

m_12 = aov(lm(formula_1, data=data %>% filter(Depth_cm==30)))#group_by(UID, Stability) %>% summarize(nSOC = mean(nSOC))))

TUKEY_12 <- TukeyHSD(x=m_12)

m_6_dt = TUKEY_2_dt(TUKEY = TUKEY_6) %>% mutate(depth = 15)

m_12_dt = TUKEY_2_dt(TUKEY = TUKEY_12) %>% mutate(depth = 30)

m_tot_dt = rbind(m_6_dt, m_12_dt)

p = TUKEY_2_plot(m_tot_dt, main = title)

}

soil_health_delta$Stability = soil_health_delta$Stability %>% toupper()

g = dpeth_tukey_plot(data = soil_health_delta,x = "Stability", y = "nSOC", title = "Rel. SOC" )+

theme(legend.position= "bottom")

g1.1 = dpeth_tukey_plot(data = soil_health_delta,x = "Stability", y = "BD", title = "Bulk denisty" )+

theme(legend.position= "bottom")

g1 = dpeth_tukey_plot(data = BD_df, x = "Zone", y = "`BD g/cc`", title = "Bulk denisty" )+

theme(legend.position= "bottom")

g2 = dpeth_tukey_plot(data = soil_health_delta,x = "Stability", y = "wsa", title = "Water stable aggragates" )+

theme(legend.position= "bottom")

g4 = dpeth_tukey_plot(data = soil_health_delta,x = "Stability", y = "OFS", title = "Overall fertility score")+

theme(legend.position= "bottom")

g3 = dpeth_tukey_plot(data = soil_health_delta,x = "Stability", y = "SHS", title = "Soil health score")+

theme(legend.position= "bottom")

g_out = ggarrange(g,g1,g2,g3,g4, ncol = 1, common.legend = TRUE, legend="bottom")

ggsave(g_out, filename = "./figures/paper_figs_231011/confidence_interval_with_Depth.jpeg", width = 3, height= 8, dpi = 700)

g_test = dpeth_tukey_plot(data = soil_health_delta,x = "Stability", y = "N.Min", title = "N.Min")+

theme(legend.position= "none")

#### compare YSZ to the enviro variables

var_in = c("claytotal.r","silttotal.r","sandtotal.r","Slope","aspect",

"log_flow_acc")

var_names = c("% clay", "% silt" ,"% sand",

"Slope", "Aspect", "Log flow accumulaiton")

list_enviros = map2(var_in, var_names, function(H,G){

dpeth_tukey_plot(data = farms_points_sf_15_all,x = "Stability", y = H,

title = G)+

theme(legend.position= "bottom")

})

order_stability = c("LS", "MS", "HS", "US")

my_comparisons = list(

c('HS', 'LS'),

c('HS', 'MS'),

c('US', 'LS'),

c('US', 'MS'),

c('HS','US'))

level_comps = list(

c("Low", "High"),

c("Low", "Medium"),

c("Medium", "High"))

stab_comps = list(

c("Stable", "Unstable"))

## 12 general stability and level figure by depth #####

Yield_level_nSOC = farms_points_sf_15_all %>%

# mutate(Stability = toupper(Stability)) %>%

ggplot(aes(level_code, nSOC))+#, col = factor(Depth_cm))) +

geom_boxplot(outlier.shape = NA)+

labs(x= '',y="Rel. SOC", col = "Depth")+

stat_compare_means(comparison=(level_comps),label = "p.signif", hide.ns= TRUE)+

theme_minimal()+ylim(-2.5, 7)#+scale_color_manual(values = c("tan", "brown"))

Yield_level_nSHS = farms_points_sf_15_all %>%

mutate(Stability = toupper(Stability))%>%

ggplot(aes(level_code, nSHS))+#, col = factor(Depth_cm))) +

geom_boxplot(outlier.shape = NA)+

labs(x= '',y="Rel. SHS", col = "Depth")+

stat_compare_means(comparison=(level_comps),label = "p.signif", hide.ns= TRUE)+

theme_minimal()+ylim(-2.5, 7)#+scale_color_manual(values = c("#DDA0DD","purple"))

Yield_stabiltiy_nSOC = farms_points_sf_15_all %>%

mutate(Stability = toupper(Stability))%>%

ggplot(aes(stability_code, nSOC))+#, col = factor(Depth_cm))) +

geom_boxplot(outlier.shape = NA)+

labs(x= '',y="Rel. SOC", col = "Depth")+

stat_compare_means(comparison=(stab_comps),label = "p.signif", hide.ns= TRUE)+

theme_minimal()+ylim(-2.5, 7)#+scale_color_manual(values = c("tan", "brown"))

Yield_stabiltiy_nSHS = farms_points_sf_15_all %>%

mutate(Stability = toupper(Stability))%>%

ggplot(aes(stability_code, nSHS))+#, col = factor(Depth_cm))) +

geom_boxplot(outlier.shape = NA)+

labs(x= '',y="Rel. SHS", col = "Depth")+

stat_compare_means(comparison=(stab_comps),label = "p.signif", hide.ns= TRUE)+

theme_minimal()+ylim(-2.5, 7)#+scale_color_manual(values = c("#DDA0DD","purple"))

Yield_SZ_SOC = farms_points_sf_15_all %>%

mutate(Stability = toupper(Stability))%>%

ggplot(aes(Stability %>% factor(levels = order_stability), nSOC))+#, col = factor(Depth_cm))) +

geom_boxplot(outlier.shape = NA)+

labs(x= '',y="Rel. SOC", col = "Depth")+

stat_compare_means(comparison=(my_comparisons),label = "p.signif", hide.ns= TRUE)+

theme_minimal()+ylim(-2.5, 7)#+scale_color_manual(values = c("#DDA0DD","purple"))

Yield_SZ_SHS = farms_points_sf_15_all %>%

mutate(Stability = toupper(Stability))%>%

ggplot(aes(Stability %>% factor(levels = order_stability), nSHS))+#, col = factor(Depth_cm))) +

geom_boxplot(outlier.shape = NA)+

labs(x= '',y="Rel. SHS", col = "Depth")+

stat_compare_means(comparison=(my_comparisons),label = "p.signif", hide.ns= TRUE)+

theme_minimal()+ylim(-2.5, 7)#+scale_color_manual(values = c("#DDA0DD","purple"))

nsoc_boxplot_out = ggarrange(Yield_stabiltiy_nSOC,Yield_level_nSOC, Yield_SZ_SOC, nrow = 1,

ncol = 3, common.legend = TRUE, legend="right", align ="h")

ggsave(filename = "./figures/paper_figs_231011//nSOC_boxplots.jpeg",

plot = nsoc_boxplot_out, width = 7.5, height= 2.5)

nSHS_boxplot_out = ggarrange( Yield_stabiltiy_nSHS,Yield_level_nSHS, Yield_SZ_SHS, nrow = 1,

ncol = 3, common.legend = TRUE, legend="right")

ggsave(filename = "./figures/paper_figs_231011//nSHS_boxplots.jpeg",

plot = nSHS_boxplot_out, width = 7.5, height= 2.5)

# relative differences between UD and LS/MS and HS and LS/MS

farms_points_sf_15_all_meean = farms_points_sf_15_all %>% group_by(Stability) %>% summarise_all(mean)

absoluteSOC_mean_HSLS_diff = farms_points_sf_15_all_meean$soc[farms_points_sf_15_all_meean$Stability=="hs"] -

farms_points_sf_15_all_meean$soc[farms_points_sf_15_all_meean$Stability=="ls"]

absoluteSOC_mean_HSLS_percentdiff = absoluteSOC_mean_HSLS_diff/farms_points_sf_15_all_meean$soc[farms_points_sf_15_all_meean$Stability=="ls"]

nSOC_mean_USLS_diff = farms_points_sf_15_all_meean$nSOC[farms_points_sf_15_all_meean$Stability=="us"] -

farms_points_sf_15_all_meean$nSOC[farms_points_sf_15_all_meean$Stability=="ls"]

nSOC_mean_USMS_diff = farms_points_sf_15_all_meean$nSOC[farms_points_sf_15_all_meean$Stability=="us"] -

farms_points_sf_15_all_meean$nSOC[farms_points_sf_15_all_meean$Stability=="ms"]

nSOC_mean_HSLS_diff = farms_points_sf_15_all_meean$nSOC[farms_points_sf_15_all_meean$Stability=="hs"] -

farms_points_sf_15_all_meean$nSOC[farms_points_sf_15_all_meean$Stability=="ls"]

nSOC_mean_HSMS_diff = farms_points_sf_15_all_meean$nSOC[farms_points_sf_15_all_meean$Stability=="hs"] -

farms_points_sf_15_all_meean$nSOC[farms_points_sf_15_all_meean$Stability=="ms"]

## 13 TUKEY analysis of all components ######

library(car)

L_test_soc =leveneTest(as.numeric(nSOC) ~ Stability_number %>% factor, farms_points_sf_15_all)

L_test_soc =leveneTest(as.numeric(nSHS) ~ Stability_number %>% factor, farms_points_sf_15_all)

##

farms_points_sf_15_all$Stability = farms_points_sf_15_all$Stability %>% toupper()

g_nsoc = dpeth_tukey_plot(data = farms_points_sf_15_all,x = "Stability", y = "nSOC", title = "Rel. SOC" )+

theme(legend.position= "bottom")

g_nSHS = dpeth_tukey_plot(data = farms_points_sf_15_all,x = "Stability", y = "nSHS", title = "Rel. SHS")+

theme(legend.position= "bottom")

g1 = dpeth_tukey_plot(data = farms_points_sf_15_all,x = "Stability", y = "BD", title = "Bulk density" )+

theme(legend.position= "bottom")

# g1 = dpeth_tukey_plot(data = BD_df %>% mutate(Zone = factor(Zone)), x = "Zone", y = "`BD g/cc`", title = "Bulk density" )+

# theme(legend.position= "bottom")

g2 = dpeth_tukey_plot(data = farms_points_sf_15_all,x = "Stability", y = "wsa", title = "Water stable aggregates" )+

theme(legend.position= "bottom")

g2.1 = dpeth_tukey_plot(data = farms_points_sf_15_all,x = "Stability", y = "CO2", title = "CO2 Burst" )+

theme(legend.position= "bottom")

g2.2 = dpeth_tukey_plot(data = farms_points_sf_15_all,x = "Stability", y = "SLAN", title = "SLAN" )+

theme(legend.position= "bottom")

g2.3 = dpeth_tukey_plot(data = farms_points_sf_15_all,x = "Stability", y = "WSOC", title = "WSOC" )+

theme(legend.position= "bottom")

g2.4 = dpeth_tukey_plot(data = farms_points_sf_15_all,x = "Stability", y = "S_Color", title = "S_Color" )+

theme(legend.position= "bottom")

g3.1 = dpeth_tukey_plot(data = farms_points_sf_15_all,x = "Stability", y = "Slope", title = "Slope" )+

theme(legend.position= "bottom")

g3.2 = dpeth_tukey_plot(data = farms_points_sf_15_all,x = "Stability", y = "aspect", title = "Aspect" )+

theme(legend.position= "bottom")

g3.3 = dpeth_tukey_plot(data = farms_points_sf_15_all,x = "Stability", y = "log_flow_acc", title = "Log flow accumulation" )+

theme(legend.position= "bottom")

g4 = dpeth_tukey_plot(data = farms_points_sf_15_all,x = "Stability", y = "OFS", title = "Overall fertility score")+

theme(legend.position= "bottom")

g5.1 = dpeth_tukey_plot(data = farms_points_sf_15_all,x = "Stability", y = "nsand", title = "Rel. sand" )+

theme(legend.position= "bottom")

g5.2 = dpeth_tukey_plot(data = farms_points_sf_15_all,x = "Stability", y = "nsilt", title = " Rel. silt" )+

theme(legend.position= "bottom")

g5.3 = dpeth_tukey_plot(data = farms_points_sf_15_all,x = "Stability", y = "nclay", title = "Rel. clay" )+

theme(legend.position= "bottom")

g5.4 = dpeth_tukey_plot(data = farms_points_sf_15_all,x = "Stability", y = "sandtotal.r", title = "Abs. sand")+

theme(legend.position= "bottom")

g5.5 = dpeth_tukey_plot(data = farms_points_sf_15_all,x = "Stability", y = "silttotal.r", title = "Abs. clay")+

theme(legend.position= "bottom")

g5.6 = dpeth_tukey_plot(data = farms_points_sf_15_all,x = "Stability", y = "claytotal.r", title = "Abs. clay")+

theme(legend.position= "bottom")

g6.1 = dpeth_tukey_plot(data = farms_points_sf_15_all,x = "level_code", y = "nSOC", title = "Yield Level v. Rel. SOC" )+

theme(legend.position= "bottom")

g6.2 = dpeth_tukey_plot(data = farms_points_sf_15_all,x = "stability_code", y = "nSOC", title = "Stability v. Rel. SOC" )+

theme(legend.position= "bottom")

g6.3 = dpeth_tukey_plot(data = farms_points_sf_15_all,x = "level_code", y = "nSHS", title = "Yield Level v. Rel. SHS" )+

theme(legend.position= "bottom")

g6.4 = dpeth_tukey_plot(data = farms_points_sf_15_all,x = "stability_code", y = "nSHS", title = "Stability v. Rel. SHS" )+

theme(legend.position= "bottom")

g_out = ggarrange(g_nsoc,g_nSHS,g1,g2,g4, ncol = 1, common.legend = TRUE, legend="bottom")

ggsave(g_out, filename = "./figures/paper_figs_231011//confidence_interval_with_Depth.jpeg", width = 3, height= 8, dpi = 700)

g_SHS = ggarrange(g2,g2.1, g2.2, g2.3, g2.4, ncol = 1, common.legend = TRUE, legend="bottom")

ggsave(g_SHS, filename = "./figures/paper_figs_231011//confidence_interval_with_Depth._shs_compt.jpeg", width = 3, height= 8, dpi = 700)

g_out = ggarrange(g3.1,g3.2,g3.3, ncol = 1, common.legend = TRUE, legend="bottom")

ggsave(g_out, filename = "./figures/paper_figs_230709/confidence_interval_with_Depth_topo_components.jpeg", width = 3, height= 6, dpi = 700)

g_out = ggarrange(g5.1,g5.2,g5.3, ncol = 1, common.legend = TRUE, legend="bottom")

ggsave(g_out, filename = "./figures/paper_figs_230709/confidence_interval_with_Depth_soil_components.jpeg", width = 3, height= 6, dpi = 700)

g_out = ggarrange(g5.4,g5.5,g5.6, ncol = 1, common.legend = TRUE, legend="bottom")

ggsave(g_out, filename = "./figures/paper_figs_230709/confidence_interval_with_Depth_abs_soil_components.jpeg", width = 3, height= 6, dpi = 700)

g_out = ggarrange(g6.1,g6.2,g6.3,g6.4, ncol = 1, common.legend = TRUE, legend="bottom")

ggsave(g_out, filename = "./figures/paper_figs_230709/confidence_interval_with_Depth_stablevel_components.jpeg", width = 4, height= 8, dpi = 700)

## 14 power analysis ####

mod= anova(lm(nSOC ~ Stability,data=farms_points_sf_15_all))

p_vals <- mod$`Pr(>F)`[1]

cat("Simulated power is:", mean(p_vals <= 0.05)*100, "%")

# Number of simulations

n_sim <- 10000

# Sample size of each group

sampsi <- c(30, 30, 30,30)

farms_points_sf_15_all %>% group_by(Stability) %>%

summarize(n=n())

# Mean of each group

mus <- c(0.368,-0.522,-0.138,0.291)

farms_points_sf_15_all %>% group_by(Stability) %>%

summarize(nSOC = mean(nSOC))

#https://stats.stackexchange.com/questions/388110/how-to-conduct-power-analysis-for-unbalanced-one-way-anova-in-r

# Standard deviation of each group (assumed to be equal!)

sds <- c(1.01, 0.976,0.733,0.935)

farms_points_sf_15_all %>% group_by(Stability) %>%

summarize(nSOC = sd(nSOC))

farms_points_sf_15_all %>%

filter(Depth_cm==15) %>% group_by(Stability) %>%

summarize(nSOC = sd(nSOC))

farms_points_sf_15_all%>%

filter(Depth_cm==30) %>% group_by(Stability) %>%

summarize(nSOC = sd(nSOC))

farms_points_sf_15_all%>%

group_by(Rep, Farm,Stability ) %>%

summarise_all(mean)%>%

group_by(Stability) %>%

summarize(nSOC = sd(nSOC))

p_vals <- NULL

# Set seed for reproducibility

set.seed(142857)

for(i in 1:n_sim) {

dat_tmp <- data.frame(

y = rnorm(sum(sampsi), mean = rep(mus, times = sampsi), sd = rep(sds, times = sampsi))

, group = factor(rep(seq_along(mus), times = sampsi))

)

mod <- anova(lm(y~group, data = dat_tmp))

p_vals[i] <- mod$`Pr(>F)`[1]

rm(dat_tmp)

}

cat("Simulated power is:", mean(p_vals <= 0.05)*100, "%")

# Install and load the package

install.packages("effectsize")

library(effectsize)

# Assuming you have a model 'my_model' from ANOVA

mod = anova(lm(nSOC ~ Stability,data=farms_points_sf_15_all%>%

group_by(Rep, Farm,Stability ) %>%

summarise_all(mean) %>% ungroup))

effect_size <- eta_squared(mod) #for small samples

omega_squared(mod)

epsilon_squared(mod)

interpret_eta_squared(0.13, rules = "cohen1992")

interpret_omega_squared(0.19, rules = "cohen1992")

mod = anova(lm(nSOC ~ Stability,data=farms_points_sf_15_all%>%

group_by(Rep, Farm,Stability ) %>%

summarise_all(mean) %>% ungroup))

effect_size <- eta_squared(mod) #for small samples

omega_squared(mod)

epsilon_squared(mod)

interpret_eta_squared(0.13, rules = "cohen1992")

interpret_omega_squared(0.19, rules = "cohen1992")

#0-15 depth

mod = anova(lm(nSOC ~ Stability,data=farms_points_sf_15_all%>%

filter(Depth_cm==15) %>% ungroup))

effect_size <- eta_squared(mod) #for small samples

omega_squared(mod)

epsilon_squared(mod)

interpret_eta_squared(0.13, rules = "cohen1992")

interpret_omega_squared(0.19, rules = "cohen1992")

#15-30 depth

mod = anova(lm(nSOC ~ Stability,data=farms_points_sf_15_all%>%

filter(Depth_cm==30)))

effect_size <- eta_squared(mod) #for small samples

omega_squared(mod)

epsilon_squared(mod)

interpret_eta_squared(0.13, rules = "cohen1992")

interpret_omega_squared(0.19, rules = "cohen1992")

## 14 plot SZ verse topography#####

g_slope = farms_points_sf_15_all %>%

mutate(Stability = toupper(Stability))%>%

ggplot(aes(factor(Stability,levels = order_stability), Slope)) +

geom_boxplot(outlier.shape = NA)+

labs(x= '',y="Slope (%)")+

stat_compare_means(comparison=(my_comparisons),label = "p.signif", hide.ns= TRUE)+

theme_minimal()

g_sflow_acc = farms_points_sf_15_all %>%

mutate(Stability = toupper(Stability))%>%

ggplot(aes(factor(Stability,levels = order_stability), log_flow_acc)) +

geom_boxplot(outlier.shape = NA)+

# ggboxplot(x = 'Stability', y ='delta_BD',# add= 'jitter',

# main=farm, order = order_stability,xlab = '', ylab= "Top's BD - sub's BD' (g/cc)")+

labs(x= '',y="Log flow accumualtion")+

stat_compare_means(comparison=(my_comparisons),

label = "p.signif", hide.ns= TRUE)+

#stat_compare_means(method = "anova")+

theme_minimal()

stab_groups = farms_points_sf_15_all %>% group_by(Stability) %>% summarise_all(mean)

stab_groups[,c("Stability", "Slope")]

stab_groups[,c("Stability", "log_flow_acc")]

ggsave(filename = "./figures/paper_figs_230709/YSZ_flow_accumulation.jpeg", plot = g_sflow_acc, width = 3, height= 2.5)

ggsave(filename = "./figures/paper_figs_230709/YSZ_slope.jpeg", plot = g_slope, width = 3, height= 2.5)

# ggsave(filename = "./figures/paper_figs_230709/YSZ_flow_accumulation_tuky.jpeg", plot = list_enviros[[6]], width = 4, height= 4)

# ggsave(filename = "./figures/paper_figs_230709/YSZ_Slope_tuky.jpeg", plot = list_enviros[[4]], width = 4, height= 4)

## 15 Management plots ####

mean_diff_hsls = farms_points_sf_15_all %>%

group_by(Farm, Stability, Depth_cm) %>%

dplyr::select( (where(is.numeric))) %>%

summarise_all(~mean(.x, na.rm=T)) %>% ungroup %>%

filter(Stability %in% c("HS","LS")) %>%

group_by(Farm, Depth_cm) %>% dplyr::select( (where(is.numeric))) %>%

summarise_all( ~.[1] - .[2]) %>% ungroup

managmentment = read_excel("C:/Users/fowler53/Michigan State University/EESBassoLab - Soil_health_variability/inputs/Management_Information_computer readable .xlsx")

mean_diff_hsls = mean_diff_hsls %>%

left_join(managmentment %>% rename(Farm = Field) %>%

dplyr::select(Farm,`2021 Crop`,Tillage,`Corn N Practice`,covercrop_TF)) %>%

mutate(`Nitrogen Fertilizer`= ifelse(`Corn N Practice`==0, "Uniform", "Variable Rate"),

Tillage = ifelse(Tillage=="Conventional", "Tillage", Tillage),

Covercrop = ifelse(covercrop_TF == 0, "No Cover crop", "Cover crop"))

g_Tillage =

mean_diff_hsls %>% #filter(Depth_cm ==30) %>%

# mutate(Stability = toupper(Stability))%>%

ggplot(aes(x = factor(Tillage), y = nSOC, col = factor(Depth_cm))) +

geom_boxplot(outlier.shape = NA)+

# ggboxplot(x = 'Stability', y ='delta_BD',# add= 'jitter',

# main=farm, order = order_stability,xlab = '', ylab= "Top's BD - sub's BD' (g/cc)")+

labs(x= '',y="HS - LS rel. SOC difference (sd)", col = "Depth")+

# stat_compare_means(comparison=(unique(mean_diff_hsls$`Corn N Practice`)),method = "t.test", label = "p.signif", hide.ns= TRUE)+

# stat_compare_means(method = "t.test")+

theme_minimal()+

scale_color_manual(values= viridis::viridis(5)[c(1,4)])

g_covercrop =

mean_diff_hsls %>% #filter(Depth_cm ==30) %>%

# mutate(Stability = toupper(Stability))%>%

ggplot(aes(x = factor(Covercrop), y = nSOC,

col = Depth_cm %>% factor)) +

geom_boxplot(outlier.shape = NA)+

# ggboxplot(x = 'Stability', y ='delta_BD',# add= 'jitter',

# main=farm, order = order_stability,xlab = '', ylab= "Top's BD - sub's BD' (g/cc)")+

labs(x= '',y="HS - LS rel. SOC difference (sd)", col = "Depth")+

# stat_compare_means(comparison=(unique(mean_diff_hsls$`Corn N Practice`)),method = "t.test", label = "p.signif", hide.ns= TRUE)+

#stat_compare_means(method = "anova")+

theme_minimal()+

scale_color_manual(values= viridis::viridis(5)[c(1,4)])

g_N_Fertilizer =

mean_diff_hsls %>%

# mutate(Stability = toupper(Stability))%>%

ggplot(aes(x = factor(`Nitrogen Fertilizer`, levels= c("Variable Rate", "Uniform")),

y = nSOC, col = Depth_cm %>% factor)) +

geom_boxplot(outlier.shape = NA)+

# ggboxplot(x = 'Stability', y ='delta_BD',# add= 'jitter',

# main=farm, order = order_stability,xlab = '', ylab= "Top's BD - sub's BD' (g/cc)")+

labs(x= '',y="HS - LS rel. SOC difference (SD)", col = 'Depth')+

# stat_compare_means(comparison=(unique(mean_diff_hsls$`Corn N Practice`)),method = "t.test", label = "p.signif", hide.ns= TRUE)+

# stat_compare_means(method = "anova")+

theme_minimal()+

scale_color_manual(values= viridis::viridis(5)[c(1,4)])

management_out = ggarrange(g_covercrop, g_Tillage, g_N_Fertilizer, nrow = 1,

ncol = 3, common.legend = TRUE, legend="right")

ggsave(filename = "./figures/paper_figs_231011//managment_boxplots.jpeg",

plot = management_out, width = 7.5, height= 2.6)

nrate = mean_diff_hsls %>% group_by(Depth_cm, `Nitrogen Fertilizer`) %>% summarize_all(median)

nrate2 = nrate[,c("Depth_cm", "nSOC", "nSHS")] %>% ungroup %>% group_by(Depth_cm) %>% summarise_all(diff)

nt = mean_diff_hsls %>% group_by(Depth_cm, Tillage) %>% summarize_all(median)

nt2 = nt[,c("Depth_cm", "nSOC", "nSHS")] %>%

ungroup %>% group_by(Depth_cm) %>% summarise_all(diff)

Covercrop = mean_diff_hsls %>% group_by(Depth_cm, Covercrop) %>% summarize_all(median)

Covercrop2 = Covercrop[,c("Depth_cm", "nSOC", "nSHS")] %>%

ungroup %>% group_by(Depth_cm) %>% summarise_all(diff)

g_Tillage_tukey = dpeth_tukey_plot(data = mean_diff_hsls,x = "Tillage", y = "nSOC", title = "Tillage" )+

theme(legend.position= "bottom")

g_nRate_tukey = dpeth_tukey_plot(data = mean_diff_hsls %>% rename(NitrogenFertilizer=`Nitrogen Fertilizer`),x = "NitrogenFertilizer", y = "nSOC", title = "Nitrogen Fertilizer" )+

theme(legend.position= "bottom")

g_Covercrop_tukey = dpeth_tukey_plot(data = mean_diff_hsls,x = "Covercrop", y = "nSOC", title = "Covercrop" )+

theme(legend.position= "bottom")

g_out = ggarrange(g_Tillage_tukey, g_nRate_tukey, g_Covercrop_tukey, ncol = 3, common.legend = TRUE, legend="bottom")

ggsave(g_out, filename = "./figures/paper_figs_230709/confidence_interval_with_Depth_managment.jpeg", width =7.5, height= 4, dpi = 700)

# S5. References

1. KSSLM 2014: Kellogg Soil Survey Laboratory Methods Manual (SSIR 42), issued 2014, Vers. 5.0, National Soil Survey Center, Lincoln NE
2. Moore, D.B., Guillard, K., Morris, T.F. and Brinton, W.F., 2019A. Correlation between Solvita labile amino-nitrogen and CO2-burst soil health tests and response to organic fertilizer in a turfgrass soil. Communications in Soil Science and Plant Analysis, 50(22), pp.2948-2959.
3. Solvita Labile Amino-Nitrogen and CO2-Burst Soil Health Tests and Response to Organic Fertilizer in a Turfgrass Soil. Communications In Soil Science and Plant Analysis <https://doi.org/10.1080/00103624.2019.1689258>
4. Brinton WF, Vallotton J (2019) Basis for Comparisons of Soil CO2 Respiration Test Procedures. Agricultural & Environmental Letters doi.org/10.2134/ael2018.10.0053.
5. Comeau, L.P., MacKinley, K., Unc, A. and Vallotton, J., 2022. Ex situ soil respiration assessment using minimally disturbed microcosms and dried–sieved soils; comparison of methods to assess soil health. Canadian Journal of Soil Science, 103(1), pp.143-151.
6. Moore, D.B., Guillard, K., Morris, T.F. and Brinton, W.F., 2019B. Predicting cool‐season turfgrass response with Solvita soil tests, Part 2: CO2–Burst carbon concentrations. Crop Science, 59(5), pp.2237-2248.
